# Impact and cost‐effectiveness of the national scale‐up of HIV pre‐exposure prophylaxis among female sex workers in South Africa: a modelling analysis

**DOI:** 10.1002/jia2.26063

**Published:** 2023-02-20

**Authors:** Jack Stone, Rutendo Bothma, Gabriela B. Gomez, Robyn Eakle, Christinah Mukandavire, Hasina Subedar, Hannah Fraser, Marie‐Claude Boily, Sheree Schwartz, Jenny Coetzee, Kennedy Otwombe, Minja Milovanovic, Stefan Baral, Leigh F. Johnson, Willem Daniel Francois Venter, Helen Rees, Peter Vickerman

**Affiliations:** ^1^ Population Health Sciences University of Bristol Bristol UK; ^2^ Wits RHI University of the Witwatersrand Johannesburg South Africa; ^3^ Department of Global Health and Development London School of Hygiene and Tropical Medicine London UK; ^4^ Office of HIV AIDS U.S. Agency for International Development (USAID) Washington DC USA; ^5^ Department of Infectious Disease Epidemiology Imperial College London London UK; ^6^ National Department of Health Pretoria South Africa; ^7^ Department of Epidemiology Johns Hopkins Bloomberg School of Public Health Baltimore Maryland USA; ^8^ Perinatal HIV Research Unit Faculty of Health Sciences University of the Witwatersrand Johannesburg South Africa; ^9^ South African Medical Research Council Cape Town South Africa; ^10^ African Potential Management Consultancy Kyalami South Africa; ^11^ School of Public Health Faculty of Health Sciences University of the Witwatersrand Johannesburg South Africa; ^12^ Centre for Infectious Disease Epidemiology and Research University of Cape Town Cape Town South Africa; ^13^ Ezintsha Faculty of Health Sciences University of the Witwatersrand Johannesburg South Africa

**Keywords:** female sex workers, HIV, mathematical modelling, pre‐exposure prophylaxis, prevention, South Africa

## Abstract

**Introduction:**

In 2016, South Africa (SA) initiated a national programme to scale‐up pre‐exposure prophylaxis (PrEP) among female sex workers (FSWs), with ∼20,000 PrEP initiations among FSWs (∼14% of FSW) by 2020. We evaluated the impact and cost‐effectiveness of this programme, including future scale‐up scenarios and the potential detrimental impact of the COVID‐19 pandemic.

**Methods:**

A compartmental HIV transmission model for SA was adapted to include PrEP. Using estimates on self‐reported PrEP adherence from a national study of FSW (67.7%) and the Treatment and Prevention for FSWs (TAPS) PrEP demonstration study in SA (80.8%), we down‐adjusted TAPS estimates for the proportion of FSWs with detectable drug levels (adjusted range: 38.0–70.4%). The model stratified FSW by low (undetectable drug; 0% efficacy) and high adherence (detectable drug; 79.9%; 95% CI: 67.2–87.6% efficacy). FSWs can transition between adherence levels, with lower loss‐to‐follow‐up among highly adherent FSWs (aHR: 0.58; 95% CI: 0.40–0.85; TAPS data). The model was calibrated to monthly data on the national scale‐up of PrEP among FSWs over 2016–2020, including reductions in PrEP initiations during 2020. The model projected the impact of the current programme (2016–2020) and the future impact (2021–2040) at current coverage or if initiation and/or retention are doubled. Using published cost data, we assessed the cost‐effectiveness (healthcare provider perspective; 3% discount rate; time horizon 2016–2040) of the current PrEP provision.

**Results:**

Calibrated to national data, model projections suggest that 2.1% of HIV‐negative FSWs were currently on PrEP in 2020, with PrEP preventing 0.45% (95% credibility interval, 0.35–0.57%) of HIV infections among FSWs over 2016–2020 or 605 (444–840) infections overall. Reductions in PrEP initiations in 2020 possibly reduced infections averted by 18.57% (13.99–23.29). PrEP is cost‐saving, with $1.42 (1.03–1.99) of ART costs saved per dollar spent on PrEP. Going forward, existing coverage of PrEP will avert 5,635 (3,572–9,036) infections by 2040. However, if PrEP initiation and retention doubles, then PrEP coverage increases to 9.9% (8.7–11.6%) and impact increases 4.3 times with 24,114 (15,308–38,107) infections averted by 2040.

**Conclusions:**

Our findings advocate for the expansion of PrEP to FSWs throughout SA to maximize its impact. This should include strategies to optimize retention and should target women in contact with FSW services.

## 1 INTRODUCTION

HIV prevalence among female sex workers (FSWs) in South Africa (SA) is high (40–72% [1, 2, 3, 4]). Although sex work is criminalized, government‐supported programmes provide HIV prevention and treatment services for FSWs [5]. In 2015, a demonstration study (Treatment And Prevention for female Sex workers or “TAPS” study) was started in Johannesburg and Pretoria to integrate HIV pre‐exposure prophylaxis (PrEP) into FSW‐targeted services [6]. This study achieved high uptake (219 FSWs initiated PrEP) but with considerable loss‐to‐follow‐up (LTFU; 71%) after 12 months [4]. In 2016, SA initiated a national programme providing PrEP for FSWs, with 20,754 PrEP initiations by 2020. Although evidence suggests that PrEP retention among FSWs was unchanged during the COVID‐19 pandemic [7], PrEP initiations did decline in 2020.

A recent review of existing modelling and cost‐effectiveness studies of PrEP summarized that too few are informed by real‐world uptake, retention and adherence data and few use cost data from actual interventions [8]. For modelling PrEP among FSWs, only one study has utilized data from a real project [9].

Utilizing data on PrEP uptake and retention from the national scale‐up of PrEP among FSWs in SA, we use modelling to estimate the “real‐world” impact and cost‐effectiveness of this national programme, including the potential effect of the COVID‐19 pandemic.

## 2 METHODS

### 2.1 Model description

We adapted a published dynamic compartmental HIV transmission model [10] to project the impact of PrEP among FSWs in SA. The model divides the population into different adult (15‐ to 49‐year‐olds) sub‐populations, including low‐risk males and females, FSWs, their clients and men who have sex with men (MSM).

People enter the model as low‐risk males or females or young MSM at a rate that balances non‐HIV deaths, ageing out of the model and reflecting population growth. A proportion are HIV positive. Low‐risk males and females can become clients and FSWs, respectively, for an average duration before returning to the low‐risk groups (Figure S1).

**Figure 1:**
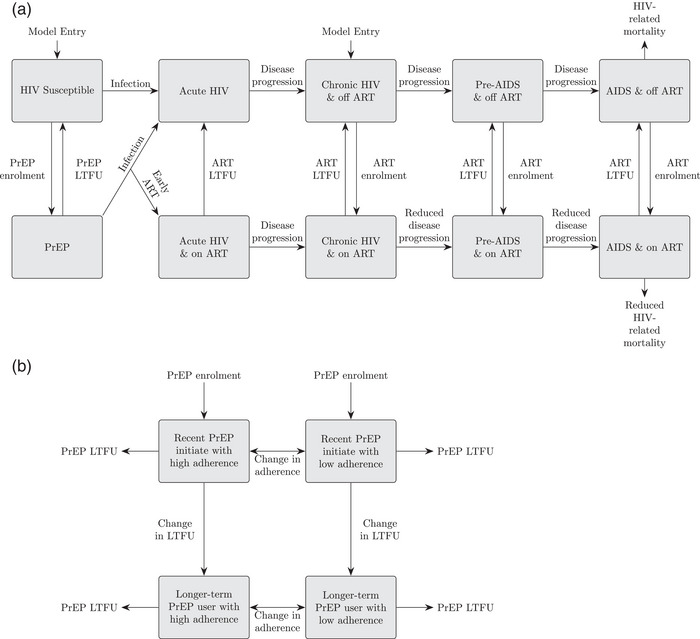
Model schematics. (a) Illustrates the stratification of the population with respect to HIV infection, antiretroviral treatment (ART) and PrEP. (b) Shows stratification of the population using PrEP (shown simplistically as one component in (a)).

The model captures sexual HIV transmission between males and females due to main, casual and commercial sexual partnerships. The model simulates HIV infection, disease progression and ART (Figure 1). ART reduces HIV‐related mortality and HIV infectivity.

HIV susceptible FSWs initiate PrEP at a time‐varying rate. Individuals first enter the recently initiated PrEP category as low or high adherers, then transitioning to the longer‐term PrEP category (justification in **parameterization** section). FSWs transition between low and high adherence. PrEP cessation rates differ by whether FSWs have recently initiated PrEP or not and by adherence level. FSWs also stop PrEP if they cease sex work or acquire HIV, there upon a proportion initiate ART immediately. The model is described in the Supplementary Materials [10].

### 2.2 Model parameterization and calibration

The model was parameterized and calibrated using data from SA, including nine FSW surveys (1997–2017 [1 – 4, 11]), two client surveys (2017/18 [12]) and five general population surveys (2002–2017 [13 – 17]). These surveys gave sub‐group‐specific estimates for sexual risk behaviours, changes in condom use, HIV prevalence and incidence, male circumcision and ART coverage and viral suppression. FSW size estimates came from a national study [18], while the client population size was estimated indirectly [10]. Table 1 summarizes data used for FSWs and their clients (full details in [10]).

**Table 1:** Summary of main prior parameter ranges and calibration data (recent estimates) used for female sex workers and their clients

	FSWs		Clients	
	Range	Source	Range	Source
Calibration data				
Size estimate (% of adult female population—2013)	0.69–0.96%	[18]	Balance commercial sex acts	
HIV prevalence (2014–2019)	49.2–61.5%	[1, 2, 3, 4, 11]	14.0–28.7%	[12] and*
ART coverage	39.1–59.3% (2014–2015)	[1, 3, 11, 66]	26.7–37.3% (2017–2018)	[12] and*
Model parameters				
Average duration of commercial sex (years)	3.2–8.1	[1, 3, 11]	1.5–3.6	[12] and*
% currently having a main partner	25.0–90.0%	[1, 3, 11]	81.0–98.0%	[12] and*
% currently having a casual partner	5.6–29.0%	[1, 3]	53.4–97.9%	[12] and*
Frequency of commercial sex partners per year^a^	100–1000	[1, 3, 11]	2.3–72.8	[12, 13, 17] and*
Frequency of main partners per year^a^	1.0–3.1	[1, 3, 13, 17]	1.0–2.9	[12] and*
Frequency of casual partners per year^a^	1.0–18.0	[1, 3, 13]	1.1–15.1	[12] and*
% of commercial sex acts that are anal^b^	0.6–9.3%	[3] and *	0.6–9.3%	[3] and *
Frequency of vaginal sex acts among main partners (per year)	24–144	[1, 3, 11]	6–144	*
Frequency of vaginal sex acts among casual partners (per partner)	0.2–8.3	[1, 3]	0.5–10.2	*
Frequency of anal sex acts among main partners (per year)	1.6–60.0	[1, 3, 11]	0–7.2	*
Frequency of anal sex acts among casual partners (per partner)	0.0–3.3	[1, 3, 11]	0.0–0.52	*
Condom use during vaginal sex commercial partner (%) ^c^	30–98.8% from 2008 onwards	[1, 3, 11, 12, 17, 67] and *	30–98.8% from 2008 onwards	[1, 3, 11, 12, 17, 67] and *
Condom use during vaginal sex with main partner (%) ^d^	13.0–44.1% from 2013 onwards	[1, 3, 67]	9.7–57.6% from 2008 onwards	[12] and *
Condom use during vaginal sex with casual partner (%) ^d^	Assumed 1.25–1.75 times higher than with main partners	[67]	32.2–86.2% from 2005 onwards	[12] and *

Note: Full details of the data used to calibrate the model and prior parameter ranges are in [10].

^a^ Among those with partners.

^b^ The rest are vaginal.

^c^ Condom use in commercial anal sex assumed to be 0.5–1 times that of commercial vaginal sex.

^d^ Due to limited data for anal sex, condom use for anal sex is assumed to be the same as for vaginal sex with main or casual partners.

* Unpublished survey of clients of FSW in Port Elizabeth.

The model was calibrated using approximate Bayesian methods [19] to population size estimates, ART coverage levels, and HIV incidence and prevalence data up to 2017. This produced 10,000 baseline model fits which were used to give the median and 95% credibility intervals (95% CrI; 2.5th–97.5th percentile range) for model projections. The baseline model calibration did not incorporate PrEP or changes due to COVID‐19. Going forward, existing rates of ART scale‐up are continued in each risk group until 90.25% ART coverage (UNAIDS 95/95/95 target) is achieved. Figures S2–S5 show that the model agrees well with available HIV epidemiological data [10].

### 2.3 PrEP parameterization

**Figure 2:**
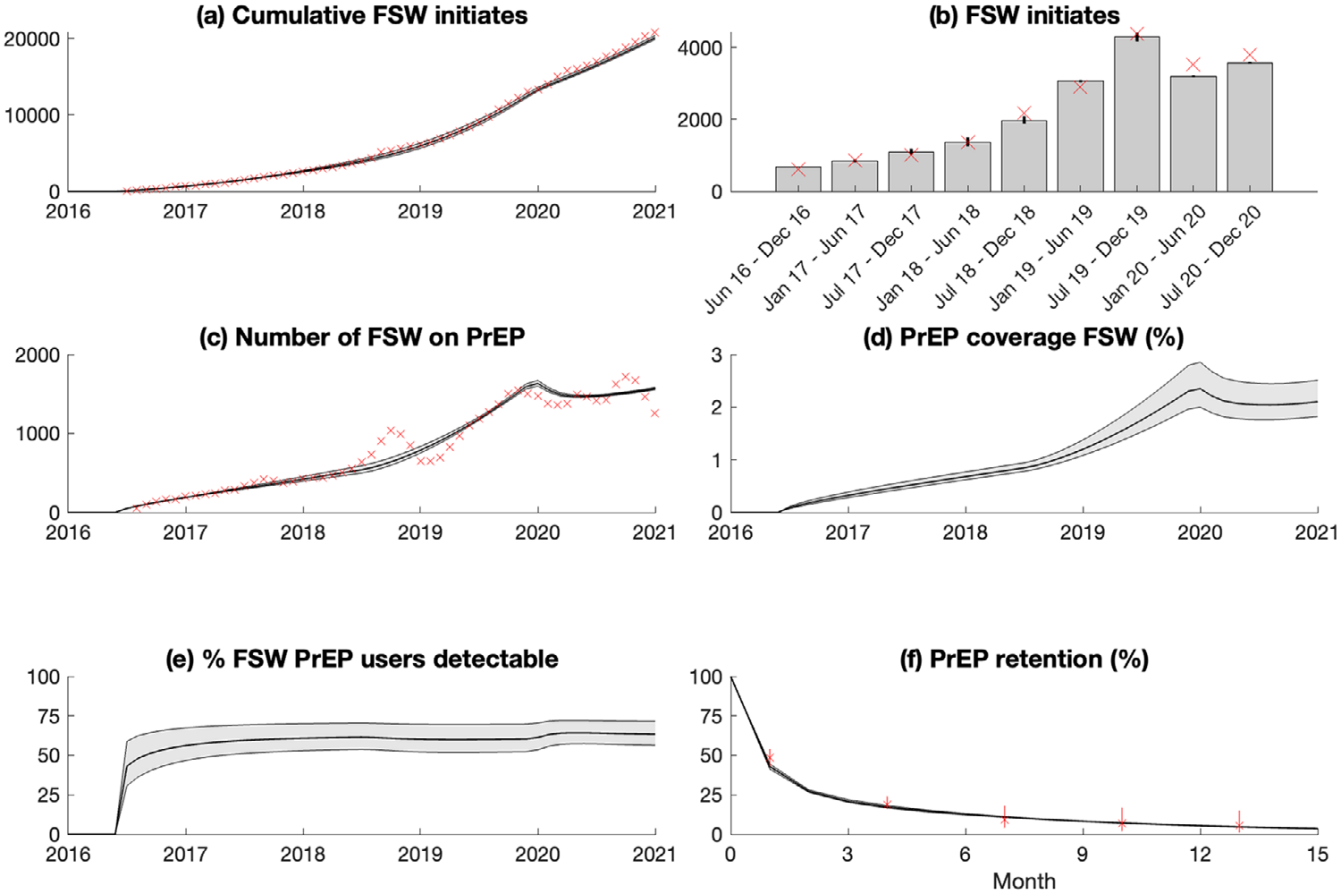
Modelled PrEP scale‐up among FSWs (black lines are median model projections and grey shading are 95% credibility intervals) and comparison with programmatic data from the national scale‐up of PrEP among FSWs (red crosses). (a) Cumulative number of PrEP initiates among FSWs. (b) Number of new PrEP initiates among FSWs over 6‐month time periods (except first period which is 7 months)—grey bars are median model projections. (c) Number of FSW currently on PrEP (only the final datapoint is used in model calibration). (d) Modelled proportion of HIV‐negative FSWs who are on PrEP. (e) Modelled proportion of FSWs who are on PrEP that have detectable drug level, that is highly adherent. (f) Proportion of FSWs retained on PrEP after different durations since initiation.

National data were available over 2016–2020 on the monthly number of FSWs initiated on PrEP and currently taking PrEP, and levels of PrEP retention (Figure 2a,c,f). These data were used to model PrEP scale‐up among FSWs, with there being 20,754 PrEP initiations over 2016–2020 and 1258 FSWs still on PrEP by December 2020. Because retention data suggested that only 48% of FSWs remained on PrEP for over 1 month, but retention improved after that, PrEP retention was modelled using two categories: recent PrEP initiates (with high LTFU rates) and longer‐term PrEP users (lower LTFU). To fit to the national data on PrEP uptake, we estimated initiation rates at five different time points (initiation rates change linearly between time points), with initiation rates remaining constant after 2020. This calibration also estimated the time after initiating PrEP that LTFU decreases, the proportion of PrEP initiates who are low adherers, and LTFU rates for low adherers and recent/longer‐term PrEP users. We assumed that LTFU was lower among FSWs with high adherence (aHR: 0.58, 95% CI: 0.40–0.85) based on TAPS data (Table S1 and Figure S7), and that individuals transition between adherence levels (Table 2).

**Table 2:** PrEP parameter values

**Parameter**	**Values**	**Source and notes**
PrEP initiation rates	Calibrated	Calibrated to total number of individuals initiating PrEP at FSW clinics in South Africa over June 2016–December 2020 (Figure 2a). Initiation rates are calibrated for: 1st June 2016, 1st July 2018, 1st December 2019, 1st February 2020, 1st January 2021 and are assumed to change linearly between these dates. These changepoints were chosen through trial and error and informed by trends in the monthly number of FSW initiates.
Proportion of FSW on PrEP who are high adherers^a^ after 2 months	Average 53.0%, range 38.0–70.4%.	Self‐reported data from a national survey of 3005 FSWs recruited from 12 sites in 2019 estimated that 67.7% (95% CI: 60.0–74.8) of 164 FSWs on PrEP were fully adherent over last 4 weeks. This is lower than levels of self‐reported adherence (80.8%, 95% CI: 76.1–85.0, averaged across 3, 6, 9 and 12 months follow‐up) achieved in the TAPS [4]. In TAPS, 88/141 (62.2%, 95% CI: 53.9–70.4) had detectable drug levels at 1‐ or 3‐month visits. We sample the proportion with detectable drugs from a normal distribution (mean 62.2% and 95% CI 53.9–70.4) which is then multiplied by a factor sampled uniformly from 0.705 (60.0/85.0)^b^ to 1.
PrEP LTFU rate among recent PrEP initiates who are low adherers^a^	Calibrated	Calibrated to National PrEP retention data for FSW (Figure 2f)
PrEP LTFU rate among long‐term PrEP users who are low adherers^a^	Calibrated	Calibrated to National PrEP retention data for FSW (Figure 2f)
Duration of time PrEP users are recent initiates	Calibrated	Calibrated to National PrEP retention data for FSW (Figure 2f)
Relative risk of LTFU among high adherers versus low adherers^a^	Lognormal distribution (0.67, 95% CI: 0.47–0.96)	Hazard ratio calculated from TAPS analyses, with high adherers defined as those with detectable drug level (see Table 4) [4].
Probability of transitioning from low adherence to high adherence^a^ within 3 months	Normal distribution (38.2%, 95% CI: 28.1–49.1)	In TAPS, transition probability from undetectable drug level to detectable over 3 months is 34/89 (38.2%, 95% CI: 28.1–49.1) [4].
Probability of transitioning from high adherence to low adherence^a^ within 3 months	Normal distribution (15.1%, 95% CI: 10.2–21.0)	In TAPS, transition probability from detectable drug level to undetectable over 3 months is 28/186 (15.1%, 95% CI: 10.2–21.0) [4].
Efficacy of PrEP for those with undetectable drug levels	0%	In TAPS, the limit of detection of drugs is 10 ng/mol. Trials with a high proportion with undetectable drugs generally had no efficacy [4, 22, 23].
Efficacy of PrEP for those with detectable drug levels	Lognormal distribution (83.8% 95% CI: 74.0–89.9)	Estimated efficacy for 100% adherence from meta‐regression of study efficacy estimates and proportion of participants adherent (determined by detectable drug level). See Supplementary Materials [21].
Proportion of PrEP users who immediately start ART when they acquire HIV	Uniform distribution (70–100%)	(Rutendo Bothma personal communication) ∼100% linkage to ART in the WITS programme (Johannesburg) with same day initiation. Assume may be as low as 70% nationally as same day initiation is not always possible.

Note: All normal and lognormal distributions are truncated to the 95% confidence intervals.

^a^ Low adherence is defined as not having detectable drug levels, whereas high adherence is defined as having detectable drug levels.

^b^ Value is produced by dividing lower CI of self‐reported adherence from national survey by upper bound of CI of self‐reported adherence in TAPs to get biggest difference.

Self‐reported data from a 2019 national survey of FSWs estimated that 67.7% (95% CI: 60.0–74.8) of FSWs on PrEP were fully adherent over the last 4 weeks, lower than the self‐reported adherence (80.8%, 95% CI: 76.1–85.0) from TAPS [4]. Plasma drug‐level testing from TAPS (limit of quantification 10.0 ng/ml, consistent with dosing in last 2–3 days [20]) suggested that 62.2% (95% CI: 53.9–70.4%) of FSWs were highly adherent, defined as having detectable drugs (80% with drug levels > = 40 ng/ml, equivalent to daily dosing [20]). Because adherence levels in the national PrEP programme are uncertain, we applied a factor reduction (with uncertainty) to the proportion of FSWs with detectable drug levels in TAPS to estimate the proportion of PrEP users in the national programme who are highly adherent. This factor reduction is sampled uniformly between 0.705 and 1 with the lower value being the lower CI of self‐reported adherence from the national FSW survey divided by the upper CI of self‐reported adherence in TAPs.

We estimated the efficacy of PrEP for a highly adherent population using data from a recent systematic review [21] (Figure S6). This was used to parameterize the efficacy of PrEP for FSWs with detectable drug levels/high adherence (79.9%; 95% CI: 67.2–87.6), while we assumed no efficacy with undetectable drug levels/low adherence [22, 23].

Based on the national FSW survey and TAPS data [4], we assumed no change in condom use while on PrEP for commercial sex, but reduced condom use for non‐commercial partnerships (OR: 0.50, 95% CI: 0.32–0.80; Supplementary Materials). For FSWs on PrEP who acquire HIV, we assumed that 70–100% initiate ART immediately based on high (∼100%) linkage to ART in Johannesburg (Rutendo Bothma, personal communication) but allowing this to be lower nationally. Table 2 summarizes the PrEP parameters.

### 2.4 Effects of COVID‐19

Based on data from KwaZulu‐Natal [24], we assumed rates of ART initiation reduced by 47.2% (95% CI 37.0–54.1%) in April 2020, recovering at a rate of 15.6% per month (95% CI 8.5–23.0). The rate of PrEP uptake among FSWs was calibrated to national data over 2020–2021, which suggested new initiates decreased in 2020 and the number of FSWs on PrEP stabilized (Figure 2b,c). In a recent survey of 343 FSWs in Gauteng and Limpopo (Rutendo Bothma, personal communication), 70% of FSWs reported reduced income during lockdowns, while 38% ceased sex work. This aligns with other studies from sub‐Saharan Africa which suggested that FSWs experienced large reductions (≥70%) in clients during the pandemic [25 – 27] and condom use with clients decreased by 16–32%. We, therefore, assumed that rates of commercial sex were 50–80% lower during lockdown periods that restricted nighttime venues [28], with condom use during commercial sex decreasing by 0–32%.

### 2.5 Impact analyses

We estimated the number and percentage of new HIV infections averted among FSWs and the overall population (15‐ to 49‐year‐olds) due to PrEP scale‐up over 2016–2020, by comparing to a counterfactual scenario without PrEP. We then estimated the impact of the following scenarios of continuing PrEP over 2021–2040 compared to a counterfactual with no PrEP over 2021–2040:

1. **Status quo**: PrEP continues at 2020 levels of initiation and LTFU (Table 2).
2. **Improved retention**: LTFU rates are halved.
3. **Increased initiation**: Initiation rates are doubled; likely involving optimizing the recruitment of FSWs in existing clinics [29].
4. **Improved initiation and retention**: (scenarios 2+3 combined).

We also estimated the impact that the COVID‐19 pandemic had on the FSW HIV epidemic and the impact of PrEP by comparing to a counterfactual that assumed no change in FSW behaviour and ART initiation in 2020, and PrEP initiations rates in 2020 increasing at the same ratio as they did over 2018–2019.

### 2.6 Cost‐effectiveness analysis

We estimated the cost‐effectiveness of the current scale‐up of PrEP over 2016–2020, with costs and disability‐adjusted life years (DALYs) tracked over 2016–2040 and PrEP ceasing in 2020 to isolate the costs and benefits resulting from PrEP provided over 2016–2020. The counterfactual assumed no PrEP over 2016–2040 to estimate the cost‐effectiveness of existing PrEP provision. The incremental cost‐effectiveness ratio (ICER) was estimated in terms of the discounted incremental costs divided by the discounted DALYs averted. The ICER was compared to a willingness‐to‐pay (WTP) threshold of 43% of GDP (US$2,582); the lowest threshold based on health opportunity costs [30]. We also estimated the net monetary benefit (NMB) of the intervention, estimated as the WTP threshold multiplied by the DALYs averted minus the overall intervention's costs. Lastly, for each of the future PrEP scenarios 2–4, we estimated the maximum additional cost per person‐year of PrEP for these addition interventions to be cost‐effective over 2021–2040 at the WTP threshold.

We used recent national estimates for the healthcare provider costs of providing PrEP to FSWs [31] (Table 3) and ART to adults in SA [32]. DALYs were estimated by applying HIV‐specific disability weights to different HIV disease stages [33] (Table 3). Costs and DALYs were discounted by 3% annually [34].

**Table 3:** Costs (in 2019 US$ from a healthcare perspective) and health utility assumptions

Parameter	Values	Notes
**Unit costs** ^a^		
Cost of PrEP initiation	US$ 25.00	Costs in 2019 US$. Cost of first year of PrEP is $134. Cost of subsequent years is $109 [31].
PrEP unit costs per year	US$ 109.00	Costs in 2019 US$ [31].
ART costs per year	US$ 265.14	Annual cost for adults is 2017/2018 US$ 249.15. This was inflated to 2019 US$ [32].
**Disability weights**		
Acute or chronic HIV infection (on/off ART)	0.078 (triangular, 0.052–0.111)	No weights so used weights for HIV/AIDS: receiving antiretroviral treatment [33].
Pre‐AIDS, off ART	0.274 (triangular, 0.183–0.377)	Weights for HIV: symptomatic, pre‐AIDS [33].
AIDS, off ART	0.582 (triangular, 0.406–0.743)	Weights for AIDS: not receiving antiretroviral treatment [33].
Pre‐AIDS or AIDS, on ART	0.078 (triangular, 0.052–0.111)	Weights for HIV/AIDS: receiving antiretroviral treatment [33].

^a^ PrEP unit costs assume that PrEP is provided at a primary health clinic alongside condom distribution, HIV testing and counselling, adherence counselling and testing for STIs. They include costs for staff, generic drugs and laboratory test costs at the screening and 3‐monthly visits. ART costs assume that ART is provided at outpatient clinics and include costs for drugs, laboratory investigations, outpatient visits, staff, infrastructure and other fixed costs.

### 2.7 Sensitivity analyses

We evaluated how the NMB of the current scale‐up of PrEP over 2016–2020 (costs and DALYs tracked to 2040) would vary if:

- Assume no transitioning between PrEP adherence levels and no difference in LTFU by adherence level.
- 80% [35] or 30% [22] of FSWs initiate PrEP as high adherers.
- PrEP drugs cost 25% less (overall PrEP cost $97.45/year instead of $109/year) or 365% more (equivalent to the non‐profit price for long‐acting injectable cabotegravir [36]; overall PrEP cost $277.80/year).
- Overall, ART costs 10% lower or 25% higher (equivalent to the maximum additional cost of injectable ART compared with current therapy for it to be cost‐effective in sub‐Saharan Africa [37]).
- Costs and DALYs discounted at 0% or 6% per year.
- Longer (40 years) or shorter (10 years) follow‐up after 2020.
- Condom use decreases during commercial sex among PrEP users as assumed for non‐commercial partnerships.
- PrEP continues over 2021–2040 instead of ceasing in 2020.
- Assume no effect of the COVID‐19 pandemic on reducing ART and PrEP uptake, commercial sex and condom use.

### 2.8 Uncertainty analyses

A regression analysis of covariance (ANCOVA) was undertaken to determine which parameter uncertainties contribute most to variability in the proportion of HIV infections averted, DALYs averted and costs averted (over 2016–2040) for existing PrEP scale‐up over 2016–2020.

## 3 RESULTS

### 3.1 Impact of existing PrEP scale‐up

Calibrated to data on the national PrEP scale‐up, projections suggest that 2.10% (95% CrI 1.82–2.51) of HIV‐negative FSWs in SA were on PrEP by 2021 (Figure 2). This PrEP scale‐up is estimated to have prevented 170 (95% CrI 115–247) or 0.45% (95% CrI 0.35–0.57) of new HIV infections among FSWs over 2016–2020 and 605 (95% CrI 444–840) or 0.045% (95% CrI 0.035–0.059) of HIV infections in the overall population. Two‐thirds of these infections averted (384, 95% CrI 265–557) were due to PrEP, the remainder being due to immediate initiation of ART if they acquire HIV while on PrEP, with 9.53 (95% CrI 6.51–13.84) person‐years of PrEP per HIV infection prevented.

The COVID‐19 pandemic likely reduced the impact of PrEP, with 18.57% (95% CrI 13.99–23.29) fewer infections averted over 2016–2020 than if PrEP initiations had increased in 2020 without the pandemic. The pandemic also reduced the HIV incidence among FSWs by 15.99% (95% CrI 9.74–21.75) during 2020 due to reductions in commercial sex.

### 3.2 Cost‐effectiveness of existing PrEP provision

Compared to a scenario with no PrEP, the national PrEP scale‐up among FSWs over 2016–2020 will result in incremental cost‐savings of US$368,000 (95% CrI –27,000 to 807,000) over 2016–2040 (due to reductions in ART costs, Table 4), with 3,890 (95% CrI 2,778–5,455) DALYs and 1,061 (95% CrI 747–1,538) HIV infections averted (Table 4). Projections suggest that for each dollar spent on PrEP among FSWs, $1.42 (95% CrI 1.03–1.99) will be saved in ART costs, with the NMB being US$10.41 million (95% CrI 7.21–14.85) by 2040. Uncertainty analyses (Figures S8 and S9) suggest that there is a 98.73% probability that the national PrEP scale‐up among FSWs will be cost‐saving and a 100% probability that it will be cost‐effective.

**Table 4:** Cost‐effectiveness of PrEP

	**No PrEP scenario**	**PrEP scenario**	**Incremental costs and DALYs**
PrEP costs (2016–2040) ($)	0 (0, 0)	824,025 (813,483, 838,200)	824,025 (813,483, 838,200)
ART costs (2016–2040) (Million $)	15,915.81 (14,526.70, 17,539.37)	15,914.62 (14,525.450, 17,537.94)	–1.19 (–1.63, –0.86)
Total costs (2016–2040) (Million $)	15,915.81 (14,526.70, 17,539.37)	15,915.44 (14,526.32, 17,538.77)	–0.37 (–0.81, –0.03)
DALYs (2016–2040)			3,890.18 (2,777.88, 5,454.82)
HIV infections (2016–2040)	4,374,143 (3,607,669, 5,241,909)	4,372,988 (3,606,908, 5,240,617)	1,061 (747, 1,538)
Mean ICER (/DALY averted)			Cost‐saving
Mean ICER (/HIV infection averted)			Cost‐saving
Net monetary benefit (Million $)			10.41

Note: Costs, DALYs and life‐years are discounted at 3% per annum.

Most variability in the proportion of HIV infections averted, DALYs averted and costs of the existing intervention were due to uncertainty in HIV transmission‐related parameters (Table S2), the efficacy of PrEP and the proportion of seroconverters that immediately start ART. Importantly, there is a strong positive association between the projected HIV incidence among FSWs and the estimated impact and costs saved by PrEP (Figure S10). These associations suggest that PrEP would cease to be cost‐saving if the HIV incidence among FSWs was <5.18 per 100 person‐years.

**Figure 3:**
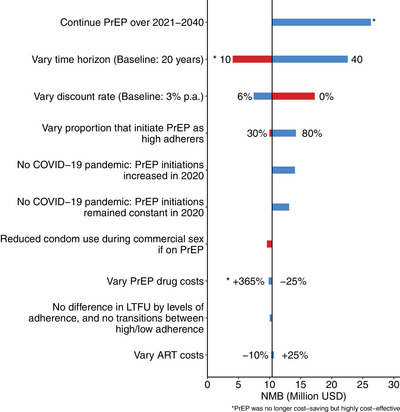
Sensitivity analyses on cost‐effectiveness projections for existing scale‐up of PrEP over 2016–2020 (benefits tracked to 2040). Bars show the mean NMB in each of the sensitivity analyses with numbers at the end of each bar being the value used in the sensitivity analysis. Red bars indicate a decrease in parameter values compared to baseline; blue bars indicate an increase in parameter values compared to baseline. The solid vertical black line shows the mean baseline NMB. Changes in the ART and PrEP costs have little effect because the NMB is dominated by the term that multiplies the WTP threshold by the DALYs averted.

In all sensitivity analyses (Figure 3), the NMB was over US$4 million and always cost‐saving unless we modelled PrEP continuing over 2021–2040, considered a shorter time horizon of 10 years (2030) or considered much higher PrEP costs (similar to injectable PrEP). These scenarios were still highly cost‐effective (ICER<US$251.23/DALY averted). Although still cost‐saving, the COVID‐19 pandemic likely reduced the NMB (by <26%) and impact of PrEP over 2016–2020, with 28.04% (95% CrI 22.53–33.30) fewer HIV infections averted by 2040.

### 3.3 Impact of future PrEP provision

**Figure 4:**
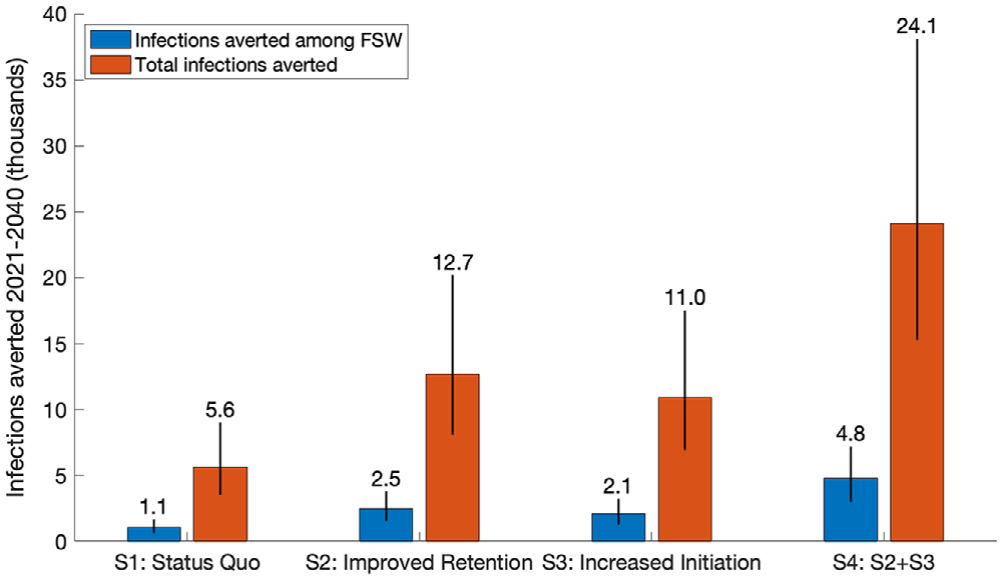
Future impact of PrEP provision among FSWs on HIV infections averted from 2021 to 2040. Scenarios considered, Status quo—PrEP continues at current levels of initiation and LTFU (S1); Improved retention—LTFU rates are halved (S2); Increased initiation—initiation rate is doubled (S3); S2+S3—Increased initiation and improved retention (S4). Bars show the median number of new HIV infections averted among FSWs (blue) or overall (red). Solid black lines show the 95% credibility intervals of projections. Numbers above these black lines show the median number of infections averted in thousands.

Over 2021–2040, maintaining status quo (2020) rates of initiation onto PrEP among FSWs will maintain PrEP coverage at 2.25% (95% CrI 1.96–2.69) of HIV‐negative FSWs, with 1.05% (95% CrI 0.83–1.30) of HIV infections being averted among FSWs over 2021–2040 and 5635 (95% CrI 3572–9036) infections being averted in the overall population, all compared to providing no PrEP to FSWs over 2021–2040 (Figure 4). If the PrEP initiation rate is doubled and LTFU is halved, then PrEP coverage would increase to 9.85% (95% CrI 8.65–11.57) of HIV‐negative FSWs. This increases impact 4.27 times (95% CrI 4.14–4.39) compared to continuing the status quo, with 24,114 (95% CrI 15,308–38,107) infections prevented in the overall population over 2021–2040; a fifth of which are among FSWs (4,805; 95% CrI 3027–7201). This equates to 4.54% (95% CrI 3.73–5.46) of HIV infections being averted among FSWs and 0.81% (95% CrI 0.58–1.19) in the overall population over 2021–2040, with 8.41 (95% CrI 5.41–12.94) person‐years of PrEP per HIV infection prevented. Doubling the initiation rate onto PrEP or halving LTFU by themselves increases impact 1.94 times (95% CrI 1.94–1.95) and 2.26 times (95% CrI 2.19–2.32), respectively, compared to the status quo scenario. Changes to PrEP delivery that double initiation or halve LTFU would be cost‐effective over 2021–2040 if the additional per person‐year costs of PrEP do not exceed US$493 or US$542, respectively. Improving both initiation and retention in combination would be cost‐effective up to an additional cost of US$542 per person‐year of PrEP.

## 4 DISCUSSION

Our modelling projects that the national PrEP scale‐up among FSWs has prevented ∼600 HIV infections over 2016–2020 and could be cost‐saving when benefits are tracked to 2040 to capture future ART cost‐savings. The population‐level impact has been limited by the low coverage of PrEP among FSWs (2.1% of HIV‐negative FSW in 2020), and so only 0.5% of new HIV infections among FSWs were prevented since 2016 and 0.05% of overall (15‐ to 49‐year‐olds) HIV infections in SA. This low impact may have been accentuated by reductions in PrEP initiations due to the COVID‐19 pandemic, which we project may have reduced infections averted by 19%. Going forward, 5600 HIV infections could be prevented over 2021–2040 with current levels of PrEP initiations, with this impact increasing four‐fold if the initiation rate of FSWs onto PrEP is doubled and LTFU is halved. Although up to $500 per person‐year of PrEP could be invested to achieve these improvements and be cost‐effective, the impact will still be modest compared to what is needed to achieve HIV elimination in SA.

### 4.1 Strengths and limitations

The main strength of our analysis focuses on utilizing real‐world national uptake and retention data on the use of PrEP among FSWs in SA [8], allowing us to estimate the real‐world population impact and cost‐effectiveness. We also used a previously validated model of HIV transmission for SA [10], which utilized comprehensive data to calibrate the model within a Bayesian framework.

Limitations include those of the previous modelling [10]. The model focused on 15‐ to 49‐year‐olds where 90% of infections occur [38]. This may have underestimated the impact and cost‐effectiveness of PrEP. Our compartmental model makes simplifying assumptions about HIV transmission across partnerships, which may overestimate levels of HIV transmission through long‐term partnerships [39], and does not capture individual‐level differences in risk behaviours or differences by age (except for MSM). However, these model simplifications should not affect our projections for FSWs, with our model also including important added detail on levels of adherence and retention for PrEP, and how they may relate. Although PrEP adherence data were unavailable from the national scale‐up, we adjusted estimates of adherence from TAPS, which may be higher than PrEP use outside of the demonstration project, to account for lower levels of self‐reported adherence from a recent national FSW survey. We also modelled adherence simply based on having detectable drug levels or not and so do not capture variability among those with detectable drug levels. However, in the TAPS study, 80% of those with detectable drug levels had levels equivalent to daily dosing, suggesting high levels of use.

Also, our model did not consider heterogeneity in risk behaviours among FSWs and was calibrated using national data and so ignores spatial differences in risk, HIV prevalence and coverage of PrEP. Our projections show that the impact and cost‐effectiveness of PrEP reduces with lower HIV incidence, suggesting that less impact will be achieved among lower‐risk FSWs or in lower‐incidence regions. Lastly, we did not consider HIV drug resistance because existing models suggest the limited impact of PrEP on drug resistance [40].

### 4.2 Comparison with other studies

Studies have previously modelled the impact and cost‐effectiveness of PrEP in different sub‐Saharan African settings and populations [8], including FSWs, with some of these considering SA [31, 41 – 49]. These studies suggest that PrEP use among FSWs can reduce transmission among FSWs, but that the overall population‐level impact may be small in generalized epidemics where FSWs may contribute less to HIV transmission [10]. In terms of cost‐effectiveness, most analyses suggest that it is cost‐effective [31, 50] or cost‐saving [43, 44] for FSWs to use PrEP in SA, with this being diminished in other settings in sub‐Saharan Africa with lower HIV incidence because less impact is achieved [51, 52]. PrEP for FSWs was found to not be cost‐saving in SA when evaluated using a static model [31] or when PrEP is scaled up over the full 20‐year evaluation period [50], which may be because both analyses did not fully capture the future prevention benefits of PrEP (similar results were found in our sensitivity analysis).

Only one previous study from sub‐Saharan Africa, which evaluated the impact of a PrEP demonstration project for FSW in Benin [9], has used real data from FSW on PrEP uptake, adherence and retention. This analysis projected that PrEP for FSWs could have a greater population‐level impact than we projected if much higher coverage levels were achieved. When similar coverage levels were assumed (9% of HIV‐negative FSW on PrEP), then a similar percentage of infections were averted in FSWs (4.7%) and overall (0.6%) over 2 years. Our modelling analyses also incorporated data on how retention may relate to adherence and evaluates the impact and cost‐effectiveness of a real‐world national PrEP programme.

## 5 CONCLUSIONS

Using real‐world data on levels of uptake, retention and adherence among FSWs in SA, our analysis gives evidence that providing PrEP for FSWs is a cost‐saving intervention in high‐incidence settings, thus advocating for further expansion of PrEP among FSWs in SA and other high prevalence settings. Several empirical studies have shown that PrEP provided on a broad scale with variable use can significantly reduce new infections [53 – 57]. Focusing efforts to provide flexible and accessible services is likely to be the best strategy going forward, with the benefits of modest increases in uptake and initiatives to reduce LTFU likely to be relatively substantial. Decriminalizing sex work could also improve access to services [58].

Reviews have highlighted pill burden and stigma associated with taking pills as barriers to PrEP adherence among FSWs [59, 60]. In SA, ART‐experienced FSWs identified social support as a valued component of pill‐taking and ensuring commitment [61], while FSWs on PrEP cited side effects, stigma and challenges accessing PrEP as main reasons for ceasing PrEP [29]. This stigma related to PrEP pills being associated with ART for people living with HIV. Our analyses of the TAPS study dataset found that FSWs connected to sex worker groups are more likely to be retained on PrEP, suggesting that targeting those already in contact with services, while also expanding these services and incorporating initiatives to manage side effects and reduce stigma, could improve the impact of PrEP, but will increase costs. Alongside effective management of side effects, providing improved education on these side effects and that these likely alleviate with time may improve retention. However, we are unaware of any effective interventions that improve PrEP retention through reducing PrEP‐related side effects or stigma, and while new long‐acting PrEP options may remove some of the stigma and barriers to adherence and retention associated with daily pill taking, their scale‐up will take time and differ in costs to oral PrEP. Although studies demonstrate a strong preference for injectable HIV prevention products or multi‐purpose technologies that also provide protection against STIs and pregnancies among women in SA, there is heterogeneity in these preferences [62, 63]. It is, therefore, important that a variety of prevention methodologies are offered, alongside ensuring adequate social support to optimize the uptake of PrEP among FSWs. Although there is a growing impetus to consider implementing family planning services with PrEP delivery for FSWs [64], the low coverage of PrEP alongside the high prevalence of unintended pregnancies among this population [65] suggests that family planning services should be integrated more broadly with FSW programming. Importantly, although PrEP is a valuable prevention tool, epidemic control among FSWs and their partners in high‐prevalence settings will only be achieved through a comprehensive prevention approach including such things as condom promotion and ART scale‐up [10].

## COMPETING INTERESTS

HF has received an honorarium from MSD unrelated to this research. PV has received unrestricted research funding from Gilead unrelated to this work.

## AUTHORS’ CONTRIBUTIONS

PV, GBG, RE and HR conceptualized the study. JS and PV developed the project and JS performed the model analyses, which were initiated by CM. PV supervised the project. PV and JS wrote the first draft of the manuscript. JS and CM reviewed the literature for data sources for the model. RB, GBG, RE, HS, SS, JC, SB, WDFV and HR collected or contributed data for the modelling. All authors contributed to data interpretation, writing the manuscript and approved the final version.

## FUNDING

Funding for this project primarily came from the Bill and Melinda Gates Foundation [OPP1084416] and the United States Agency for International Development [AID‐674‐A‐12‐00034]. Additionally, funding was also provided by a supplement to the Johns Hopkins University Center for AIDS Research, a National Institutes of Health (NIH) funded programme (P30AI094189) with support specifically from the Office of AIDS Research (OAR). The original model development received support from the Linkages across the Continuum of HIV Services for Key Populations Affected by HIV project (LINKAGES, Cooperative Agreement AID‐OAA‐A‐14‐00045) and the parent study HIV Prevention 2.0 (HP2): Achieving an AIDS‐Free Generation in Senegal (AID‐OAA‐A‐13‐00089). The supplement, LINKAGES and HP2 received support from the United States Agency for International Development (USAID) and the U.S. President's Emergency Plan for AIDS Relief (PEPFAR).  JC was supported by the South African Medical Research Council through its Division of Research Capacity Development under the Research Capacity Development Initiative (RCDI) programme. Funding was also received in part by the Wellcome Trust [214204/A/18/Z]. SB and SS were funded by R01MH121161‐01A1 and R01NR016650‐04S1.

## DISCLAIMER

The content is solely the responsibility of the authors and does not necessarily represent the official views of any of the funding agencies. GBG is currently employed by Sanofi Pasteur. Sanofi Pasteur did not provide funding for this work and had no role in study design, data collection, data analysis, data interpretation or writing of the report. RE is currently employed by USAID which occurred after the original project ended. This article was made possible by the support of the American people through the United States Agency for International Development (USAID) under the U.S. President's Emergency Plan for AIDS Relief (PEPFAR). The contents of this article are the sole responsibility of the authors and do not necessarily reflect the views of USAID, PEPFAR or the United States Government. This work was supported, in whole or in part, by the Bill & Melinda Gates Foundation [OPP1084416]. Under the grant conditions of the Foundation, a Creative Commons Attribution 4.0 Generic License has already been assigned to the Author Accepted Manuscript version that might arise from this submission.

## Supporting information

Supporting Information

## Data Availability

Model code will be made available following publication. The code will be shared with researchers who provide a methodologically sound proposal approved by JS and PV. Proposals should be directed to jack.stone@bristol.ac.uk and peter.vickerman@bristol.ac.uk; requesters will need to sign a data access agreement.
