# ERRATUM

**DOI:** 10.1002/jia2.26082

**Published:** 2023-04-17

In the article [1], the affiliation of the author, Marie‐Claude Boily, is incorrect.

The incorrect affiliation is:

^5^Department of Infectious Disease Epidemiology, Imperial College London, London, UK

The correct affiliation should be:

^14^MRC Centre for Global Infectious Disease Analysis, School of Public Health, Imperial College London, London, UK
