# Supplementary material for: Impact and cost‐effectiveness of the national scale‐up of HIV pre‐exposure prophylaxis among female sex workers in South Africa: a modelling analysis
Source: J Int AIDS Soc. 2023 Feb 20;26(2):e26063. doi: 10.1002/jia2.26063 (PMC9939943; doi:10.1002/jia2.26063)
Supplement: Supplementary file 1 — Supporting Information [file JIA2-26-e26063-s001.docx]

**Supplementary Materials to: Impact and cost-effectiveness of the national scale-up of HIV pre-exposure prophylaxis among female sex workers in South Africa: A modelling analysis**

Jack Stone, Rutendo Bothma, Gabriela B. Gomez, Robyn Eakle, Christinah Mukandavire, Hasina Subedar, Hannah Fraser, Marie-Claude Boily, Sheree Schwartz, Jenny Coetzee, Kennedy Otwombe, Minja Milovanovic, Stefan Baral, Leigh F. Johnson, Willem Daniel Francois Venter, Helen Rees, Peter Vickerman

Table of Contents

[Model description 2](#_Toc125013518)

[Model equations 4](#_Toc125013519)

[Condom use assumptions 9](#_Toc125013520)

[Model Calibration 9](#_Toc125013521)

[Model Cross-validation 10](#_Toc125013522)

[Estimation of DALYs Averted 10](#_Toc125013523)

[PrEP Efficacy assumptions 15](#_Toc125013524)

[Results 16](#_Toc125013525)

[References 21](#_Toc125013526)

# Model description

We used a previously published dynamic HIV transmission model (Mukandavire, Walker et al. 2018, Stone, Mukandavire et al. 2021). The model considers adults (15-49 years), and divides the population into six sub-populations: low-risk females ($i=1$) and males $(i=2$), clients ($i=3$), FSW ($i=4$), young MSM ($<30 years, i=5$) and older MSM ($\geq30 years, i=6$) (Figure 1 shows the model schematic). Low-risk individuals are defined as people that are not MSM and do not report commercial sex.

People enter the modelled population when they become sexually active, at a rate $\theta$ that balances non-HIV deaths and reflects population growth, with a proportion $p$ entering into the low-risk female group, $\left( 1-p \right)(1-p_{2})$ entering the low-risk male group and $\left( 1-p \right)p_{2}$ entering the young MSM group. A proportion ($\Phi_{1}$ for low-risk females and $\Phi_{2}$ for low-risk males and MSM) of those entering the model are HIV positive. Low-risk females become FSW at a rate $\kappa$ and stay as FSW for duration $1/\gamma$ years. Similarly, clients transition from the low-risk male population at rate $z$. Clients remain as clients for a period of $1/g$ years before returning to the low-risk male group. Conversely, MSM move from the young to the older MSM group at a rate $\zeta,$ where they remain until death or ageing out of the model.

The model captures HIV transmission among the sub-populations through vaginal and anal sexual intercourse (VI and AI, respectively) between all males and females, and AI within the MSM group (Supplementary Figure 1). The model stratifies the population with respect to HIV infection and disease progression such that for each sub-population$i$, there is uninfected ($S_{i}$), acute infection ($E_{i}$), chronic infection off ART($I_{i}$) or on ART ($I_{i}^{t}$), Pre-AIDS infection off ART ($P_{i}$) or on ART ($P_{i}^{t}$), and AIDS off ART ($A_{i})$ or on ART ($A_{i}^{t})$, with $i=1,2...6$. There are additional states for FSW who are on PrEP ($Z_{4}^{n}:$n=1 denotes recent PrEP initiate with low PrEP adherence; n=2 denotes longer term PrEP user with low adherence; n=3 denotes recent PrEP initiate with high PrEP adherence; n=4 denotes longer term PrEP user with high PrEP adherence)and for FSW and low risk-females with acute infection who are on ART ($E_{i}^{t})$. Upon infection, susceptible individuals move to the acute stage of HIV infection before progressing to the chronic, pre-AIDS and AIDS phases of infection at fixed rates ($\eta_{1}$for acute to chronic, $\eta_{2}$for chronic to pre-AIDS and $\eta_{3}$for pre-AIDS to AIDS). In the chronic, pre-AIDS and AIDS stages, individuals are recruited onto ART at a rate $\omega_{i}$ (time dependent and differs by risk group). While on ART, HIV-related mortality is reduced by a factor $\varphi$ compared to what it was in the chronic stage. Individuals on ART can be lost to follow-up at a rate $\sigma_{i}$ (which differs by risk group), after which they can be re-recruited onto ART. Individuals with AIDS experience a HIV-related mortality rate $\delta$ which is lowered by a factor $\varphi$if on ART. All sub-populations also experience non-HIV related death and exit the model with rates $\mu_{i}$, which incorporates both non-HIV related death rates and ageing out of the model.

The model incorporates HIV transmission due to main, casual and commercial sexual partnerships. Commercial partnerships can only occur between FSW and their clients, while main and casual partnerships between men only occur amongst the two MSM groups. All other main and casual partnerships between male and females can occur between individuals from all groups, including MSM. The risk of HIV transmission for a particular individual is related to the HIV prevalence of their sexual partners, with the HIV transmission risk being elevated by a factor $\nu$ if they are in the acute stage of infection, and reduced by a factor $\alpha$ if they are on ART, all compared to the transmission risk for the chronic stage. Transmission risk is also related to the average frequency of sex acts (denoted by ${\Psi_{ijh}}^{k})$ for different types of partnerships and between risk groups $i$ and $j$ (with ${\Psi_{ijh}}^{k}={\Psi_{jih}}^{k}$ to ensure they balance) , where $k$ denotes the type of sexual partner (main, casual or commercial) and $h$ denotes the type of sexual act (VI or AI). HIV transmission is reduced through condom use by a factor $\left( 1-\varepsilon\pi_{ijh}^{k} \right),$ where $\varepsilon$is the efficacy of condom use and $\pi_{ijh}^{k}$ is the average consistency of condom use reported by those in risk groups $i$ and $j$ (with $\pi_{ijh}^{k}=\pi_{jih}^{k}$ to ensure they balance). The consistency of condom use is assumed to be time dependent and varies depending on the type of partnership. The model assumes a proportion $\xi$ of males are circumcised, with the model assuming these males have a reduced risk of HIV acquisition, modelled by a factor (1-$\xi\vartheta$), where $\vartheta$ is the efficacy of circumcision.

HIV susceptible FSWs can initiate PrEP at a time varying rate ($\alpha$).All individuals start PrEP as a recent initiate to PrEP with an initial proportion being low adherers ($q$) and the remainder ($1-q$) being high adherers. Individuals transition between low and high adherence at constant rates ($\tau_{LH}$ for low to high and $\tau_{HL}$ for high to low). Individuals also transition from being recent initiates to longer term PrEP users at a constant rate ($\tau_{c}$). FSWs cease PrEP at constant rates that differ between PrEP state ($\chi_{n}$), or if they cease sex work or become infected. A proportion ($\delta$) of these newly infected FSWs initiate ART immediately. The risk of HIV acquisition for FSW on PrEP with high adherence is reduced by a factor ($1-\nu$), where $\nu$ is the efficacy of PrEP. The model assumes no efficacy for FSW who have low adherence.

# Model equations

Low risk female population

$$\frac{dS_{1}}{dt}=\left( 1-\Phi_{1} \right)p\theta+\gamma(S_{4}+Z_{4}^{1}+Z_{4}^{2}+Z_{4}^{3}+Z_{4}^{4})-{(\Lambda}_{1}^{m}+\Lambda_{1}^{c})S_{1}-(\kappa+\mu_{1})S_{1}$$

$$\frac{dE_{1}}{dt}={(\Lambda}_{1}^{m}+\Lambda_{1}^{c})S_{1}+\gamma E_{4}-(\kappa+\eta_{1}+\mu_{1})E_{1}$$

$$\frac{dI_{1}}{dt}=\Phi_{1}p\theta+\eta_{1}E_{1}+\sigma_{1}I_{1}^{t}+\gamma I_{4}-\left( \kappa+\omega_{1}+\mu_{1}+\eta_{2} \right)I_{1}$$

$$\frac{dP_{1}}{dt}=\eta_{2}I_{1}+\sigma_{1}P_{1}^{t}+\gamma P_{4}-\left( \kappa+\omega_{1}+\mu_{1}+\eta_{3} \right)P_{1}$$

$$\frac{dA_{1}}{dt}=\eta_{3}P_{1}+\sigma_{1}A_{1}^{t}+\gamma A_{4}-\left( \kappa+\omega_{1}+\mu_{1}+\delta\right)A_{1}$$

$$\frac{dE_{1}^{t}}{dt}={\gamma E}_{4}^{t}-\left( \kappa+\eta_{1}+\mu_{1}+\sigma_{4} \right)E_{1}^{t}$$

$$\frac{dI_{1}^{t}}{dt}= \eta_{1}E_{1}^{t}+\omega_{1}I_{1}+\gamma I_{4}^{t}-\left( \kappa+\sigma_{1}+\varphi\eta_{2}+\mu_{1} \right)I_{1}^{t}$$

$$\frac{dP_{1}^{t}}{dt}=\varphi\eta_{2}I_{1}^{t}+\omega_{1}P_{1}+\gamma P_{4}^{t}-\left( \kappa+\sigma_{1}+\varphi\eta_{3}+\mu_{1} \right)P_{1}^{t}$$

$$\frac{dA_{1}^{t}}{dt}= \varphi\eta_{3}P_{1}^{t}+\omega_{1}A_{1}+\gamma A_{4}^{t}-\left( \kappa+\sigma_{1}+\varphi\delta+\mu_{1} \right)A_{1}^{t}$$

Low risk male population

$$\frac{dS_{2}}{dt}=\left( 1-\Phi_{2} \right)(1-p)(1-p_{2})\theta+gS_{3}-{(\Lambda}_{2}^{m}+\Lambda_{2}^{c})S_{2}-(z+\mu_{2})S_{2}$$

$$\frac{dE_{2}}{dt}={(\Lambda}_{2}^{m}+\Lambda_{2}^{c})S_{2}+gE_{3}-(z+\eta_{1}+\mu_{2})E_{2}$$

$$\frac{dI_{2}}{dt}=\Phi_{2}\left( 1-p \right)(1-p_{2})\theta+\eta_{1}E_{2}+\sigma_{2}I_{2}^{t}+gI_{3}-\left( z+\omega_{2}+\mu_{2}+\eta_{2} \right)I_{2}$$

$$\frac{dP_{2}}{dt}=\eta_{2}I_{2}+\sigma_{2}P_{2}^{t}+gP_{3}-\left( z+\omega_{2}+\mu_{2}+\eta_{3} \right)P_{2}$$

$$\frac{dA_{2}}{dt}=\eta_{3}P_{2}+\sigma_{2}A_{2}^{t}+gA_{3}-\left( z+\omega_{2}+\mu_{2}+\delta\right)A_{2}$$

$$\frac{dI_{2}^{t}}{dt}= \omega_{2}I_{2}+gI_{3}^{t}-\left( z+\sigma_{2}+\varphi\eta_{2}+\mu_{2} \right)I_{2}^{t}$$

$$\frac{dP_{2}^{t}}{dt}=\varphi\eta_{2}I_{2}^{t}+\omega_{2}P_{2}+gP_{3}^{t}-\left( z+\sigma_{2}+\varphi\eta_{3}+\mu_{2} \right)P_{2}^{t}$$

$$\frac{dA_{2}^{t}}{dt}= \varphi\eta_{3}P_{2}^{t}+\omega_{2}A_{2}+gA_{3}^{t}-\left( z+\sigma_{2}+\varphi\delta+\mu_{2} \right)A_{2}^{t}$$

Client population

$$\frac{dS_{3}}{dt}=zS_{2}-{(\Lambda}_{3}^{m}+\Lambda_{3}^{c})S_{3}-(g+\mu_{3})S_{3}$$

$$\frac{dE_{3}}{dt}={(\Lambda}_{3}^{m}+\Lambda_{3}^{c})S_{3}+zE_{2}-(g+\eta_{1}+\mu_{3})E_{3}$$

$$\frac{dI_{3}}{dt}=\eta_{1}E_{3}+\sigma_{3}I_{3}^{t}+zI_{2}-\left( g+\omega_{3}+\mu_{3}+\eta_{2} \right)I_{3}$$

$$\frac{dP_{3}}{dt}=\eta_{2}I_{3}+\sigma_{3}P_{3}^{t}+zP_{2}-\left( g+\omega_{3}+\mu_{3}+\eta_{3} \right)P_{3}$$

$$\frac{dA_{3}}{dt}=\eta_{3}P_{3}+\sigma_{3}A_{3}^{t}+zA_{2}-\left( g+\omega_{3}+\mu_{3}+\delta\right)A_{3}$$

$$\frac{dI_{3}^{t}}{dt}= \omega_{3}I_{3}+zI_{2}^{t}-\left( g+\sigma_{3}+\varphi\eta_{2}+\mu_{3} \right)I_{3}^{t}$$

$$\frac{dP_{3}^{t}}{dt}=\varphi\eta_{2}I_{3}^{t}+\omega_{3}P_{3}+zP_{2}^{t}-\left( g+\sigma_{3}+\varphi\eta_{3}+\mu_{3} \right)P_{3}^{t}$$

$$\frac{dA_{3}^{t}}{dt}= \varphi\eta_{3}P_{3}^{t}+\omega_{3}A_{3}+zA_{2}^{t}-\left( g+\sigma_{3}+\varphi\delta+\mu_{3} \right)A_{3}^{t}$$

FSW population

$$\frac{dS_{4}}{dt}=\kappa S_{1}-{(\Lambda}_{4}^{m}+\Lambda_{4}^{c}+\Lambda_{4}^{co})S_{4}-(\gamma+\mu_{4}+\alpha)S_{4}+\chi_{L}L_{4}+\chi_{H}H_{4}$$

$$\frac{dZ_{4}^{1}}{dt}=q\alpha S_{4}-{(\Lambda}_{4^{z}}^{m}+\Lambda_{4^{z}}^{c}+\Lambda_{4^{z}}^{co})Z_{4}^{1}-(\gamma+\mu_{4}+\tau_{\mathrm{LH}}+\tau_{c}+\chi_{1})Z_{4}^{1}+ \tau_{\mathrm{HL}}Z_{4}^{2}$$

$$\frac{dZ_{4}^{2}}{dt}=(1-q)\alpha S_{4}-{(1-\nu)(\Lambda}_{4^{z}}^{m}+\Lambda_{4^{z}}^{c}+\Lambda_{4^{z}}^{co})Z_{4}^{2}-(\gamma+\mu_{4}+\tau_{\mathrm{HL}}+\tau_{c}+\chi_{2})Z_{4}^{2}+ \tau_{\mathrm{LH}}Z_{4}^{1}$$

$$\frac{dZ_{4}^{3}}{dt}= \tau_{c}Z_{4}^{1}-{(\Lambda}_{4^{z}}^{m}+\Lambda_{4^{z}}^{c}+\Lambda_{4^{z}}^{co})Z_{4}^{3}-(\gamma+\mu_{4}+\tau_{\mathrm{LH}}+\chi_{3})Z_{4}^{3}+ \tau_{\mathrm{HL}}Z_{4}^{4}$$

$$\frac{dZ_{4}^{4}}{dt}=\tau_{c}Z_{4}^{2}-{(1-\nu)(\Lambda}_{4^{z}}^{m}+\Lambda_{4^{z}}^{c}+\Lambda_{4^{z}}^{co})Z_{4}^{4}-(\gamma+\mu_{4}+\tau_{\mathrm{HL}}+\chi_{4})Z_{4}^{4}+ \tau_{\mathrm{LH}}Z_{4}^{3}$$

$$\frac{dE_{4}}{dt}={(\Lambda}_{4}^{m}+\Lambda_{4}^{c}+\Lambda_{4}^{co})S_{4}+\left( 1-\delta\right){(\Lambda}_{4}^{m}+\Lambda_{4}^{c}+\Lambda_{4}^{co}){(L}_{4}+\left( 1-\nu\right)H_{4})+\kappa E_{1}-\left( \gamma+\eta_{1}+\mu_{4} \right)E_{4}+\sigma_{4}E_{4}^{t}$$

$$\frac{dI_{4}}{dt}=\eta_{1}E_{4}+\sigma_{4}I_{4}^{t}+\kappa I_{1}-\left( \gamma+\omega_{4}+\mu_{4}+\eta_{2} \right)I_{4}$$

$$\frac{dP_{4}}{dt}=\eta_{2}I_{4}+\sigma_{4}P_{4}^{t}+\kappa P_{1}-\left( \gamma+\omega_{4}+\mu_{4}+\eta_{3} \right)P_{4}$$

$$\frac{dA_{4}}{dt}=\eta_{3}P_{4}+\sigma_{4}A_{4}^{t}+\kappa A_{1}-\left( \gamma+\omega_{4}+\mu_{4}+\delta\right)A_{4}$$

$$\frac{dE_{4}^{t}}{dt}=\delta{(\Lambda}_{4^{z}}^{m}+\Lambda_{4^{z}}^{c}+\Lambda_{4^{z}}^{co})(Z_{4}^{1}+Z_{4}^{3}+\left( 1-\nu\right)(Z_{4}^{2}+Z_{4}^{4}))-\left( \gamma+\eta_{1}+\mu_{4}+\sigma_{4} \right)E_{4}^{t}$$

$$\frac{dI_{4}^{t}}{dt}= \eta_{1}E_{4}^{t}+\omega_{4}I_{4}+\kappa I_{4}^{t}-\left( \gamma+\sigma_{4}+\varphi\eta_{2}+\mu_{4} \right)I_{4}^{t}$$

$$\frac{dP_{4}^{t}}{dt}=\varphi\eta_{2}I_{4}^{t}+\omega_{4}P_{4}+\kappa P_{1}^{t}-\left( \gamma+\sigma_{4}+\varphi\eta_{3}+\mu_{4} \right)P_{4}^{t}$$

$$\frac{dA_{4}^{t}}{dt}= \varphi\eta_{3}P_{4}^{t}+\omega_{4}A_{4}+\kappa A_{1}^{t}-\left( \gamma+\sigma_{4}+\varphi\delta+\mu_{4} \right)A_{4}^{t}$$

MSM population-young

$$\frac{dS_{5}}{dt}=\left( 1-\Phi_{2} \right)(1-p)p_{2}\theta+gS_{3}-{(\Lambda}_{5}^{m}+\Lambda_{5}^{c}+\Lambda_{5*}^{m}+\Lambda_{5*}^{c})S_{2}-(\zeta+\mu_{5})S_{5}$$

$$\frac{dE_{5}}{dt}={(\Lambda}_{5}^{m}+\Lambda_{5}^{c}+\Lambda_{5*}^{m}+\Lambda_{5*}^{c})S_{5}-(\zeta+\eta_{1}+\mu_{5})E_{5}$$

$$\frac{dI_{5}}{dt}=\Phi_{2}\left( 1-p \right)p_{2}\theta+\eta_{1}E_{5}+\sigma_{5}I_{5}^{t}-\left( \zeta+\omega_{5}+\mu_{5}+\eta_{2} \right)I_{5}$$

$$\frac{dP_{5}}{dt}=\eta_{2}I_{5}+\sigma_{5}P_{5}^{t}-\left( \zeta+\omega_{5}+\mu_{5}+\eta_{3} \right)P_{5}$$

$$\frac{dA_{5}}{dt}=\eta_{3}P_{5}+\sigma_{5}A_{5}^{t}-\left( \zeta+\omega_{5}+\mu_{5}+\delta\right)A_{5}$$

$$\frac{dI_{5}^{t}}{dt}= \omega_{5}I_{5}-\left( \zeta+\sigma_{5}+\varphi\eta_{2}+\mu_{5} \right)I_{5}^{t}$$

$$\frac{dP_{5}^{t}}{dt}=\varphi\eta_{2}I_{5}^{t}+\omega_{5}P_{5}-\left( \zeta+\sigma_{5}+\varphi\eta_{3}+\mu_{5} \right)P_{5}^{t}$$

$$\frac{dA_{5}^{t}}{dt}= \varphi\eta_{3}P_{5}^{t}+\omega_{5}A_{5}-\left( \zeta+\sigma_{5}+\varphi\delta+\mu_{5} \right)A_{5}^{t}$$

MSM population-older

$$\frac{dS_{6}}{dt}=\zeta S_{5}-{(\Lambda}_{6}^{m}+\Lambda_{6}^{c}+\Lambda_{6*}^{m}+\Lambda_{6*}^{c})S_{6}-(\mu_{6})S_{6}$$

$$\frac{dE_{6}}{dt}={(\Lambda}_{6}^{m}+\Lambda_{6}^{c}+\Lambda_{6*}^{m}+\Lambda_{6*}^{c})S_{6}+\zeta E_{5}-(\eta_{1}+\mu_{6})E_{6}$$

$$\frac{dI_{6}}{dt}=\eta_{1}E_{6}+\sigma_{6}I_{6}^{t}+\zeta I_{5}-\left( \omega_{6}+\mu_{6}+\eta_{2} \right)I_{6}$$

$$\frac{dP_{6}}{dt}=\eta_{2}I_{6}+\sigma_{6}P_{6}^{t}+\zeta P_{5}-\left( \omega_{6}+\mu_{6}+\eta_{3} \right)P_{6}$$

$$\frac{dA_{6}}{dt}=\eta_{3}P_{6}+\sigma_{6}A_{6}^{t}+\zeta A_{5}-\left( \omega_{6}+\mu_{6}+\delta\right)A_{6}$$

$$\frac{dI_{6}^{t}}{dt}= \omega_{6}I_{6}+\zeta I_{5}^{t}-\left( \sigma_{2}+\varphi\eta_{2}+\mu_{6} \right)I_{6}^{t}$$

$$\frac{dP_{6}^{t}}{dt}=\varphi\eta_{2}I_{6}^{t}+\omega_{6}P_{6}+\zeta P_{5}^{t}-\left( \sigma_{6}+\varphi\eta_{3}+\mu_{6} \right)P_{6}^{t}$$

$$\frac{dA_{6}^{t}}{dt}= \varphi\eta_{3}P_{6}^{t}+\omega_{6}A_{6}+\zeta A_{5}^{t}-\left( \sigma_{6}+\varphi\delta+\mu_{6} \right)A_{6}^{t}$$

For females, we allow the sexual behaviour of males with females to determine who the females have sex with and how many partners they have. (A more detailed derivation can be found in (Mukandavire, Walker et al. 2018)). The FOI $\Lambda_{i}^{k}$ for the low-risk female population due to their main ($k=m$) and casual ($k=c$) partners is as follows:

$$\Lambda_{1}^{k}=\lambda_{1}\frac{\rho_{1}^{k}}{N_{1}}\sum_{j=2,3,5,6} {[\beta}_{xv}\left( 1-\varepsilon\pi_{1jv}^{k} \right)\Psi_{1jv}^{k}+\beta_{xa}\left( 1-\varepsilon\pi_{1ja}^{k} \right)\Psi_{1ja}^{k}]n_{j}^{k}N_{j}B_{j}$$

With $B_{j}$as the HIV prevalence of the population they are having sex with, which also accounts for the cofactors that increase or decrease HIV transmission risk due to the HIV acute phase $E_{j}$ ($\nu_{E})$ or the pre-AIDS and AIDS stages ($\nu_{A})$, or if on HIV treatment $T_{j}$ ($\alpha)$, such that

$$B_{j}=\frac{\nu_{E}E_{j}+I_{j}+\nu_{A}P_{j}+\left( 1-\alpha\right)(I_{j}^{t}+\nu_{A}P_{j}^{t}+\nu_{A}A_{j}^{t})}{N_{j}}$$

Where $N_{j}$ is the total population for each sub-group $j$.

The FOI for the low-risk male population due to their main $(k=m$) and casual ($k=c$) partners is

$$\Lambda_{2}^{k}=\lambda_{2}{(1-\vartheta\xi)n}_{2}^{k}\sum_{j=1 or 4} {[\beta}_{yv}\left( 1-\varepsilon\pi_{2jv}^{k} \right)\Psi_{2jv}^{k}+\beta_{ya}\left( 1-\varepsilon\pi_{2ja}^{k} \right)\Psi_{2ja}^{k}]\rho_{j}^{k}B_{j}$$

The force of infection for clients due to their main, casual ($k=m,c$) and commercial partners are given as:

$$\Lambda_{3}^{k}=\lambda_{3}(1-\vartheta\xi)n_{3}^{k}\sum_{j=1 or 4} {[\beta}_{yv}\left( 1-\varepsilon\pi_{3jv}^{k} \right)\Psi_{3jv}^{k}+\beta_{ya}\left( 1-\varepsilon\pi_{3ja}^{k} \right)\Psi_{3ja}^{k}]\rho_{j}^{k}B_{j}$$

$$\Lambda_{3}^{co}=\lambda_{3}n_{3}^{co}{[\beta}_{yv}\left( 1-\varepsilon\pi_{34v}^{co} \right)(1-p_{a}^{co})+\beta_{ya}\left( 1-\varepsilon\pi_{34a}^{c} \right)p_{a}^{co}](1-\vartheta\xi)B_{4},$$

Where $p_{a}^{co}$ is the proportion of commercial sex acts that are anal.

The FOI for female sex workers not on PrEP is similarly

$$\Lambda_{4}^{k}=\lambda_{4}\frac{\rho_{4}^{k}}{N_{4}}\sum_{j=2,3,5,6} {{[\beta}_{xv}\left( 1-\varepsilon\pi_{4jv}^{k} \right)\Psi_{4jv}^{k}+\beta_{xa}\left( 1-\varepsilon\pi_{4ja}^{k} \right)\Psi_{4ja}^{k}]n}_{j}^{k}N_{j}B_{j}$$

$$\Lambda_{4}^{co}=\lambda_{4}{n_{4}^{co}[\beta}_{xv}\left( 1-\varepsilon\pi_{43v}^{co} \right)(1-p_{a}^{co})+\beta_{xa}\left( 1-\varepsilon\pi_{43a}^{c} \right)p_{a}^{co}]B_{3}$$

The FOI for female sex workers on PrEP is

$$\Lambda_{4^{z}}^{k}=\lambda_{4}\frac{\rho_{4}^{k}}{N_{4}}\sum_{j=2,3,5,6} {{[\beta}_{xv}\left( 1-\varepsilon\pi_{4^{z}jv}^{k} \right)\Psi_{4jv}^{k}+\beta_{xa}\left( 1-z\varepsilon\pi_{4^{z}ja}^{k} \right)\Psi_{4ja}^{k}]n}_{j}^{k}N_{j}B_{j}$$

$$\Lambda_{4^{z}}^{co}=\lambda_{4}{n_{4}^{co}[\beta}_{xv}\left( 1-\varepsilon\pi_{4^{z}3v}^{co} \right)(1-p_{a}^{co})+\beta_{xa}\left( 1-\varepsilon\pi_{4^{z}3a}^{c} \right)p_{a}^{co}]B_{3}$$

Which accounts for changes in condom use if on PrEP (e.g. through $\pi_{4^{z}3v}^{co})$

Men who have sex with men are assumed to have main and casual partnerships with women from the low risk female population and FSWs as well as other MSM. The FOI for young MSM due to their main and casual ($k=m,c$) partnerships with females is:

$$\Lambda_{5}^{k}=\lambda_{5}(1-\vartheta\xi)n_{5}^{k}\sum_{j=1 or 4} {[\beta}_{yv}\left( 1-\varepsilon\pi_{5jv}^{k} \right)\Psi_{5jv}^{k}+\beta_{ya}\left( 1-\varepsilon\pi_{5ja}^{k} \right)\Psi_{5ja}^{k}]\rho_{j}^{k}B_{j}$$

For MSM sexual intercourse with their male sexual partners, we denote the receptive AI and insertive AI HIV transmission probability as $\beta_{jrec}$ and $\beta_{jins}$ respectively where $j=5,6$ and subscript *rec* and *ins* are for receptive and insertive anal intercourse between MSM. The asterisk (*) show parameters related to MSM with their male partners. Then, the FOI for young MSM due to their main and casual ($k=m,c$) partnerships with other MSM is:

$$\Lambda_{5*}^{k}=\lambda_{5}\frac{{(\beta}_{5rec}+\beta_{5ins})}{2}(1-\frac{\vartheta\xi}{2})n_{5*}^{k}\sum_{j=5 or 6} \left( 1-\varepsilon\pi_{5j*}^{k} \right){(\Psi}_{5j*}^{k})\rho_{j*}^{k}B_{j}$$

Where $\rho_{5*}^{k}$is the probability of mixing to form MSM male sexual partnerships with young or older MSM, and is given by $\rho_{5*}^{k}=n_{5*}^{k}N_{5}/(n_{5*}^{k}N_{5}+n_{6*}^{k}N_{6})$ and $\rho_{6*}^{k}=n_{6*}^{k}N_{6}/(n_{5*}^{k}N_{5}+n_{6*}^{k}N_{6})$

Similarly, the FOI for older MSM due to their female main and casual ($k=m,c$) partners

$$\Lambda_{6}^{k}=\lambda_{6}(1-\vartheta\xi)n_{6}^{k}\sum_{j=1 or 4} {[\beta}_{yv}\left( 1-\varepsilon\pi_{6jv}^{k} \right)\Psi_{6jv}^{k}+\beta_{ya}\left( 1-\varepsilon\pi_{6ja}^{k} \right)\Psi_{6ja}^{k}]\rho_{j}^{k}B_{j}$$

And due to their main and casual ($k=m,c$) partnerships with other MSM

$$\Lambda_{6*}^{k}=\lambda_{6}\frac{{(\beta}_{6rec}+\beta_{6ins})}{2}(1-\frac{\vartheta\xi}{2})n_{6*}^{k}\sum_{j=5 or 6} {\left( 1-\varepsilon\pi_{6j*}^{k} \right){(\Psi}_{6j*}^{k})\rho}_{j*}^{k}B_{j}$$

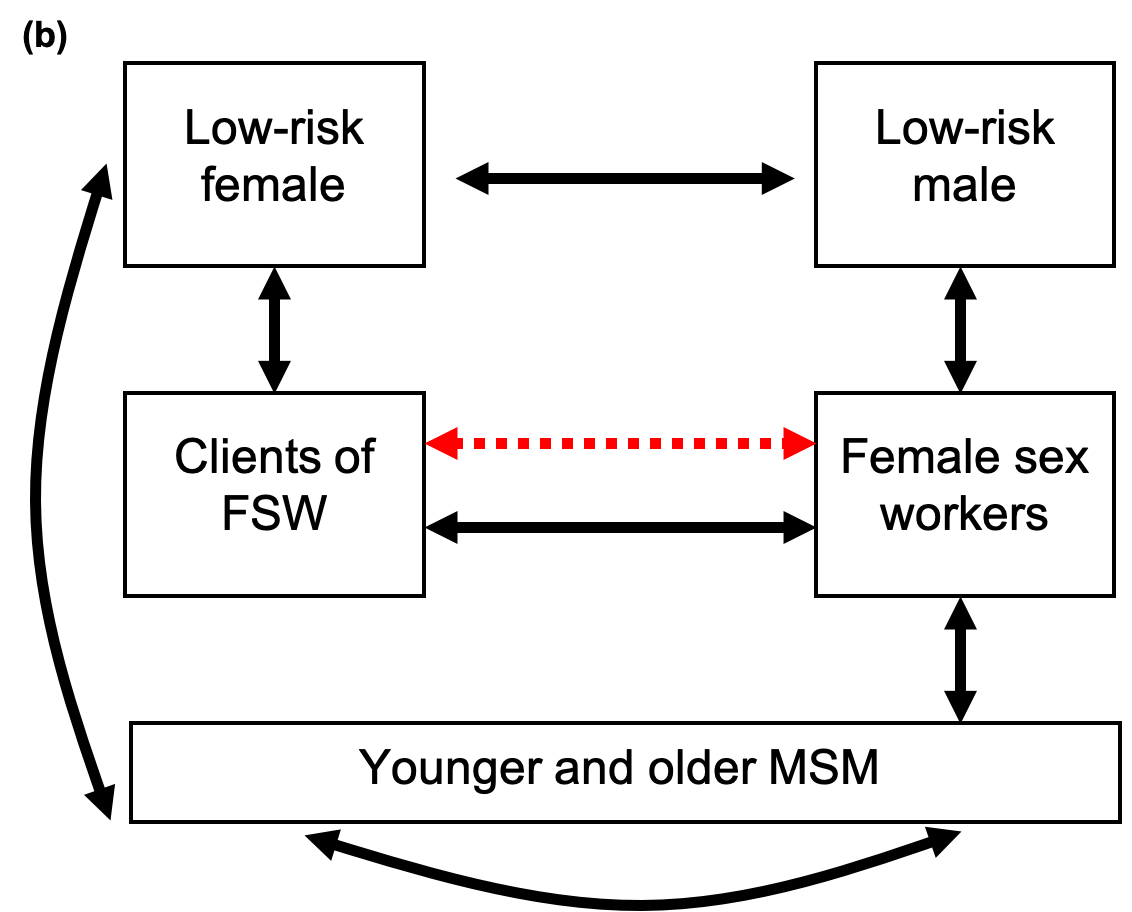


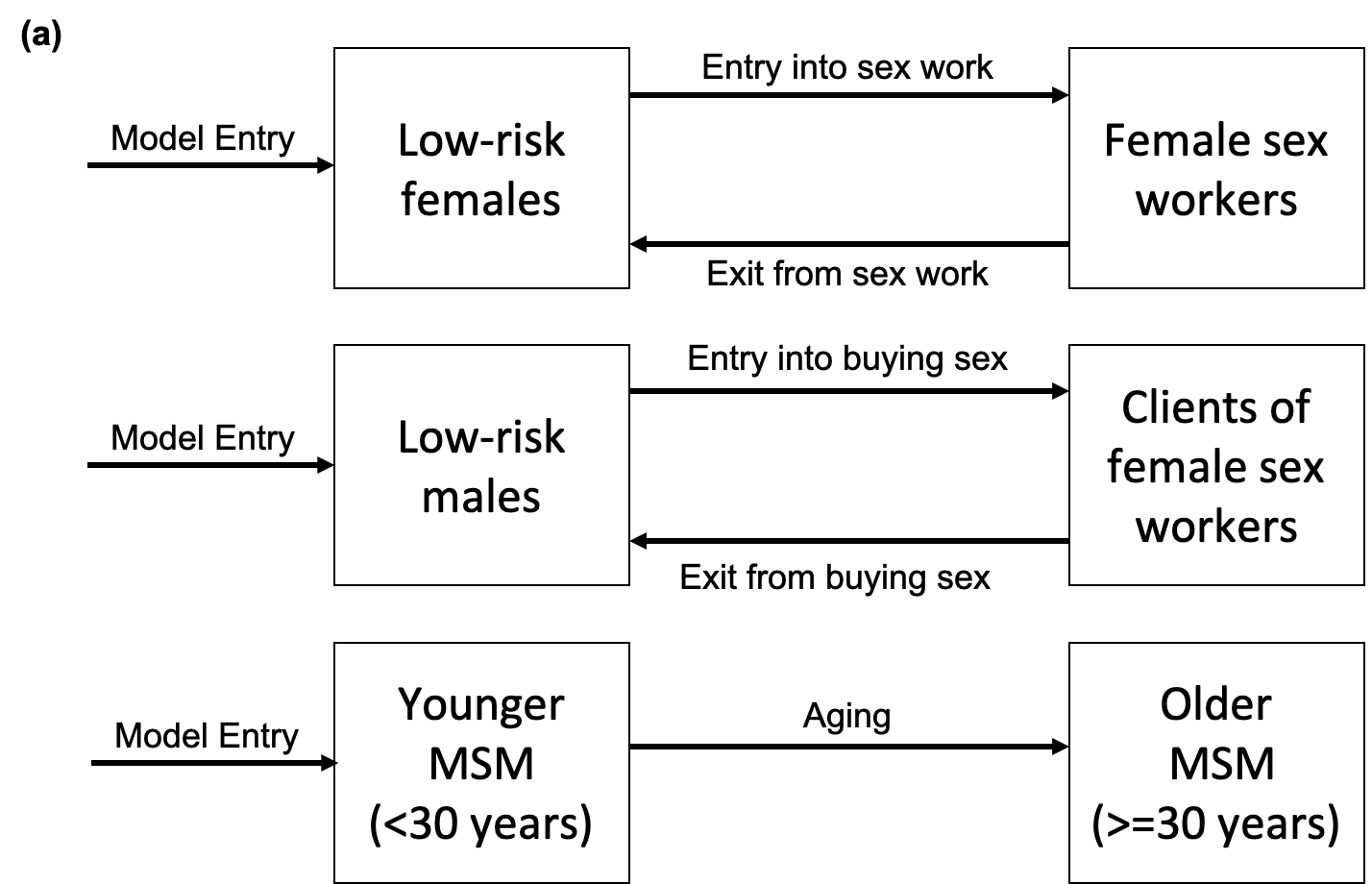


**Supplementary Figure 1: Model schematics** illustrating the (a) movement of individuals in and out of different sub-populations (b) sexual interactions which can result in HIV transmission among low-risk female, male, female sex workers, their clients and men who have sex with men. Red dashed arrow in (b) denotes commercial sex and all other arrows denote sex with main and casual partners. FSW denotes female sex worker; MSM denotes men who have sex with men.

# Condom use assumptions

*FSW – changes in condom use if on PrEP*

In the TAPS study(Eakle, Gomez et al. 2017)*,* little change over time was reported in consistent condom due to PrEP use with main, casual or commercial partners. At baseline, before initiating PrEP, 33%, 64% and >99% of FSWs reported consistent condom use with main partners, casual and commercial partners, respectively. Averaging across follow-up visits at 3, 6, 9 and 12 months, the proportion of visits in which FSW reported consistent condom use were 36% for main partners, 77% for casual partners and >99% for commercial partners. In a recent national survey of FSW(Jaffer, Christofides et al. 2022), similar proportions of HIV-negative FSWs on PrEP (97.8%) or not using PrEP (96.1%) self-reported using a condom at last commercial sex act. In contrast, HIV-negative FSWs on PrEP (23/164 - 14.02%) were less likely (OR:0.50, 95%CI 0.32-0.80) to self-report always using condoms with their main partners in the past month than those not on PrEP (237/969 - 24.46%). There was limited data on condom use with casual partner in this study. In our models, we assumed that there was no change in condom use while on PrEP for commercial sex, but that condom use decreases for main and casual partnerships while on PrEP as found in the national survey (OR:0.50, 95%CI 0.32-0.80).

# Model Calibration

The model was calibrated using an approximate Bayesian computation Sequential Monte Carlo (ABC SMC) method,(Toni, Welch et al. 2009) which accounts for uncertainty in the calibration data and parameters and ranks different model runs by their goodness of fit. The ABC method was used to calibrate the model to total population and KP size estimates, KP size estimates (FSW in 2013, MSM in 2005), ART coverage levels among all adults in 2010 and in different sub-groups in 2018, HIV incidence estimates from the HSRC surveys for the adult population, HIV prevalence among different groups: adult males and females (15-49 years) from the HSRC surveys (2005, 2012 and 2017); FSWs in 2000 (38.6-58.8%) and 2016 (45.5-68.0%); young MSM in 2009 (13.2-56.5%) and all MSM in 2016 (20.0-58.2%). Calibration ranges were defined for each estimate to be the 95% confidence interval (CI) of an estimate. For HIV prevalence among FSW and MSM, where there were multiple estimates at similar timepoints, the calibration range was defined to be the minimum lower 95%CI to the maximum upper 95%CI across these estimates. Goodness of fit for each parameter set was then defined as the sum of the absolute differences (defined as 0 if model projections lie within the calibration range) on the log scale between the lower or upper (whichever is closer to the model projections) value of the calibration ranges and the corresponding model projections.

The ABC SMC begins with 10,000 parameter sets sampled from prior distributions using Latin Hypercube sampling, which are then successively perturbed to improve their goodness of fit. At each subsequent iteration, parameter sets are resampled from the previous iteration and perturbed, until 10,000 parameter sets are obtained which each fit the data at least as well as the best 75% parameter sets from the previous iteration. The ABC continues each time the parameter sets better fit the data, until successive iterations no longer improve the goodness of the fits. The ABC routine produced a set of 10,000 baseline model fits which were used to give the median and 95% credibility intervals (95%CrI; 2.5^th^ to 97.5^th^ percentile range) for all model projections. Baseline model fits are shown in supplementary figures 3-6.

# Model Cross-validation

In all model runs, HIV prevalence among clients falls within the range of prevalences observed in Port Elizabeth and Klerksdorp, whilst 78.5% of runs fall within the range of the previous HIV incidence estimate among FSW and 46.1% of runs fall within the range of all 3 HIV incidence estimates among MSM.

# Estimation of DALYs Averted

To estimate DALYS averted by PrEP, we first calculated for each year, the difference in years lived with disease between the Intervention scenario (PrEP over 2016-2020) and the counterfactual scenario (No PrEP over 2016-2040). For both scenarios, the years lived with disease was calculated by weighting the person years in each model compartment by disability weights (values in Table 3) and summing over model compartments. We then calculated the difference in years of life lost by calculating the difference in the total number of person years between the Intervention and counterfactual scenario. DALYs averted for each year were then calculated by summing the difference in years lived with disease and the difference in life years lost. Finally, the overall DALYs averted were calculated by summing the DALYs averted across years, applying discount rates where appropriate.

**Supplementary Figure 2**. A comparison of median and 95% credibility intervals from baseline model fits (black line and shaded area) with HIV prevalence estimates for (a) overall adult population, (b) adult male and (c) adult female general population, (d) female sex workers (FSWs), (e) their clients, and (f) men who have sex with men (MSM). Red points and whiskers show data with 95% confidence intervals used for model calibration, and black points show cross validation data not used in model calibration but shown to compare with model projections.

**Supplementary Figure 3**. A comparison of median and 95% credibility intervals from baseline model fits (black line and shaded area) with HIV incidence estimates for (a) overall adult population, (b) adult male and (c) adult female general population, (d) female sex workers (FSWs), (e) their clients, and (f) men who have sex with men (MSM). Black points show cross validation data not used in model calibration but shown to validate the model projections. Note that the scale for the y-axes are different for figures (d) and (f).

**Supplementary Figure 4:** A comparison of model fits with HIV prevalence estimates (among all MSM) for (a) younger men who have sex with men (MSM), and (b) older MSM. Continuous black line shows median projections from all the model fits, with the grey shaded areas showing 95% credibility intervals. Red points and whiskers show regional HIV prevalence data estimates **among all MSM** (**not for each age strata**) with 95% confidence intervals. Most available MSM surveys are heavily weighted towards young MSM (e.g. In South African RDS studies of MSM, the median proportion of MSM who are aged <25 is 70%) and so it is to be expected that the modelled HIV prevalence among older MSM, who have been at risk of acquiring HIV for longer, will be greater than HIV prevalence data estimates among all MSM. Indeed, previous modelling(Johnson, Mulongeni et al. 2018) has suggested that RDS studies under-estimate the exceptionally high HIV prevalence rates in South African MSM because of over-sampling of younger MSM.


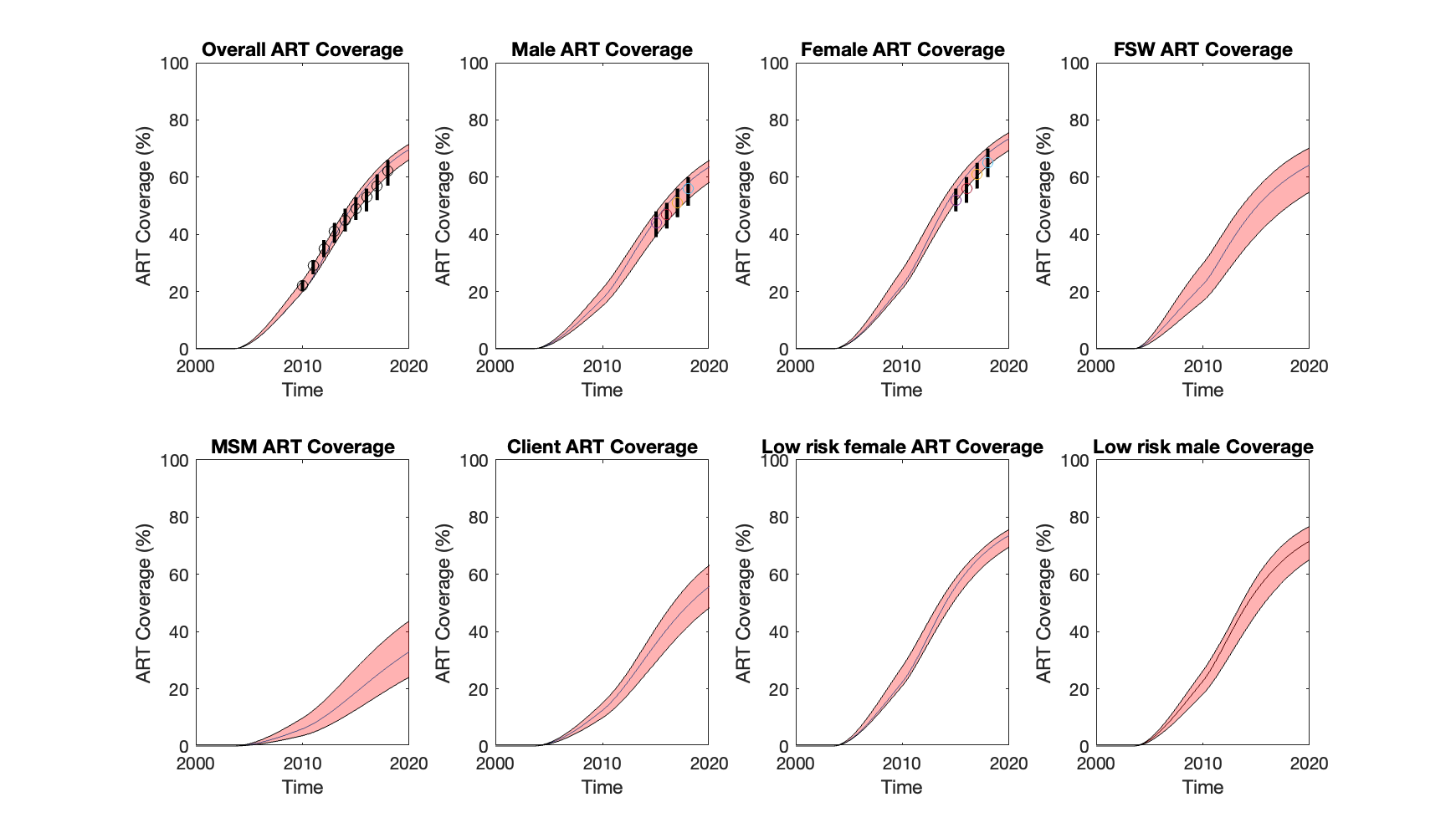


**Supplementary Figure 5:** Modelled ART trend for female sex workers (FSW), men who have sex with men (MSM) and low risk females and males. Continuous black line indicates median projections from all the baseline model fits with pink shaded areas showing 95% credibility intervals. Vertical black lines show UNAIDS estimates.

# PrEP Efficacy assumptions

Using data from a recent systematic review of PrEP efficacy studies that showed study efficacy was closely related with overall study adherence (mainly measured as the proportion of participants with detectable drug level)(Chou, Evans et al. 2019), we undertook a meta-regression analysis to estimate the efficacy of PrEP for a fully adherent population (supplementary figure 2). This gave an estimated efficacy of PrEP of 79.9% (95% CI: 67.2-87.6) when fully adherent. Because there are concerns that PrEP efficacy may be lower among females than males for reasons other than adherence(Janes, Corey et al. 2018), we performed a similar meta-regression analyses using female data only(Janes, Corey et al. 2018). This gave an estimated efficacy of PrEP of 82.5% (95%CI: 58.1-92.7) when fully adherent, which was similar to (but with more uncertainty) the estimate obtained using data for both males and females. Because of the similarity of these estimates, we used the more precise estimate (based on Chou et al.) to parameterise the efficacy of PrEP for FSWs with detectable drug levels (79.9%; 95% CI: 67.2-87.6), while we conservatively assumed no efficacy for FSWs with undetectable drug levels, in line with results of the VOICE and FEMPrEP trials(Van Damme, Corneli et al. 2012, Marrazzo, Ramjee et al. 2015).

**Supplementary Figure 6:** PrEP efficacy versus adherence for trials measuring adherence based on the presence of detectable tenofovir on drug level testing.

# Results

**Supplementary Table 1:** Factors associated with PrEP loss-to-follow-up. Model 1 includes drug level as a binary variable – detectable compared to undetectable. Model 2 includes drug level as a continuous variable.

|  | **Model 1** | | **Model 2** | |
| --- | --- | --- | --- | --- |
|  | aHR | P-value | aHR | P-value |
| Detectable TNF | 0.58 (0.40-0.85) | 0.005 | - | - |
| Per log10 increase in TNF | - |  | 0.75 (0.61-0.92) | 0.007 |
| Connected with sex worker groups (e.g. Sisonke or SWEAT) | 0.51 (0.26-1.00) | 0.049 | 0.51 (0.26-0.99) | 0.045 |
| >=30 years old | 0.72 (0.49-1.09) | 0.103 | 0.72 (0.49-1.07) | 0.103 |

**Supplementary Figure 7:** Kaplan-Meier analysis of retention in PrEP stratified by whether have detectable emtricitabine and tenofovir disoproxyl fumarate drug level.


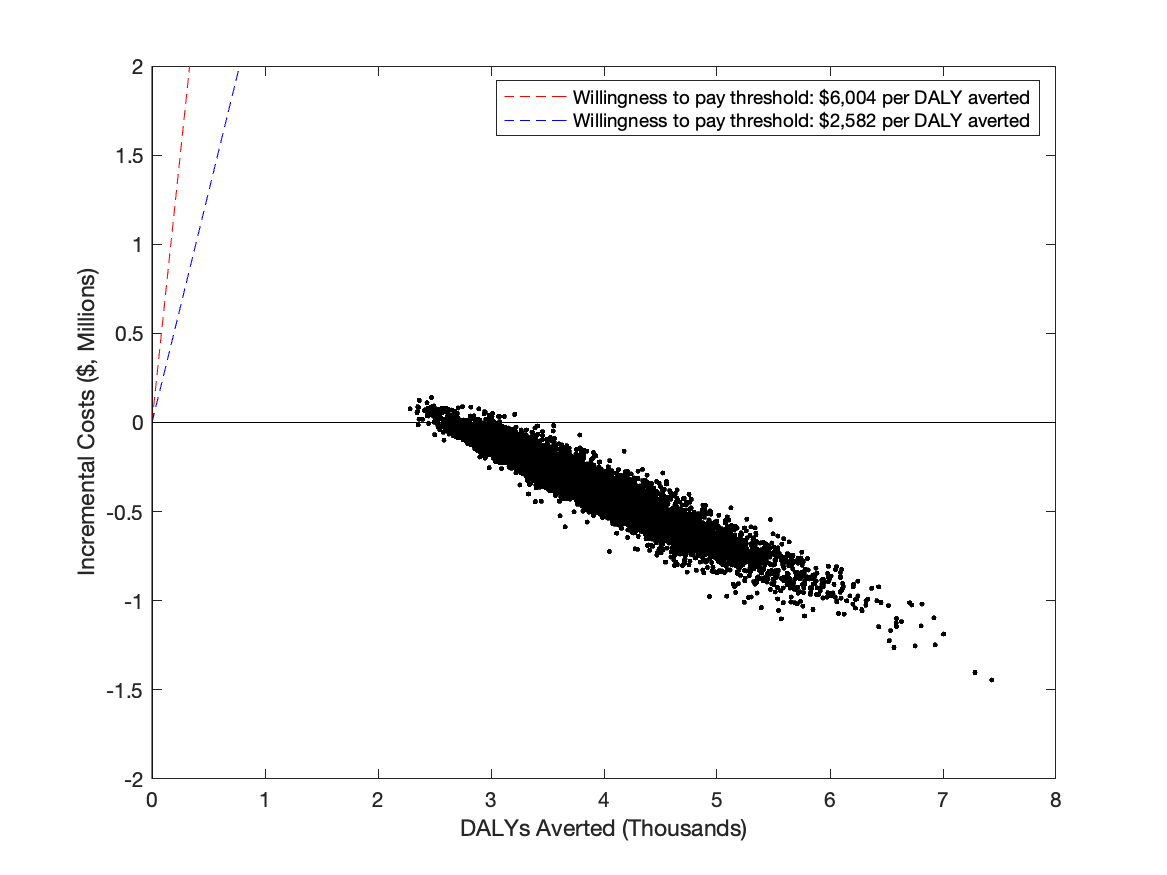


**Supplementary Figure 8:** *Cost-effectiveness plane for the Baseline cost-effectiveness analysis. Black points represent each model run. Dashed lines show the WTP threshold of 1xGDP (red) and 0.43xGDP (blue).*


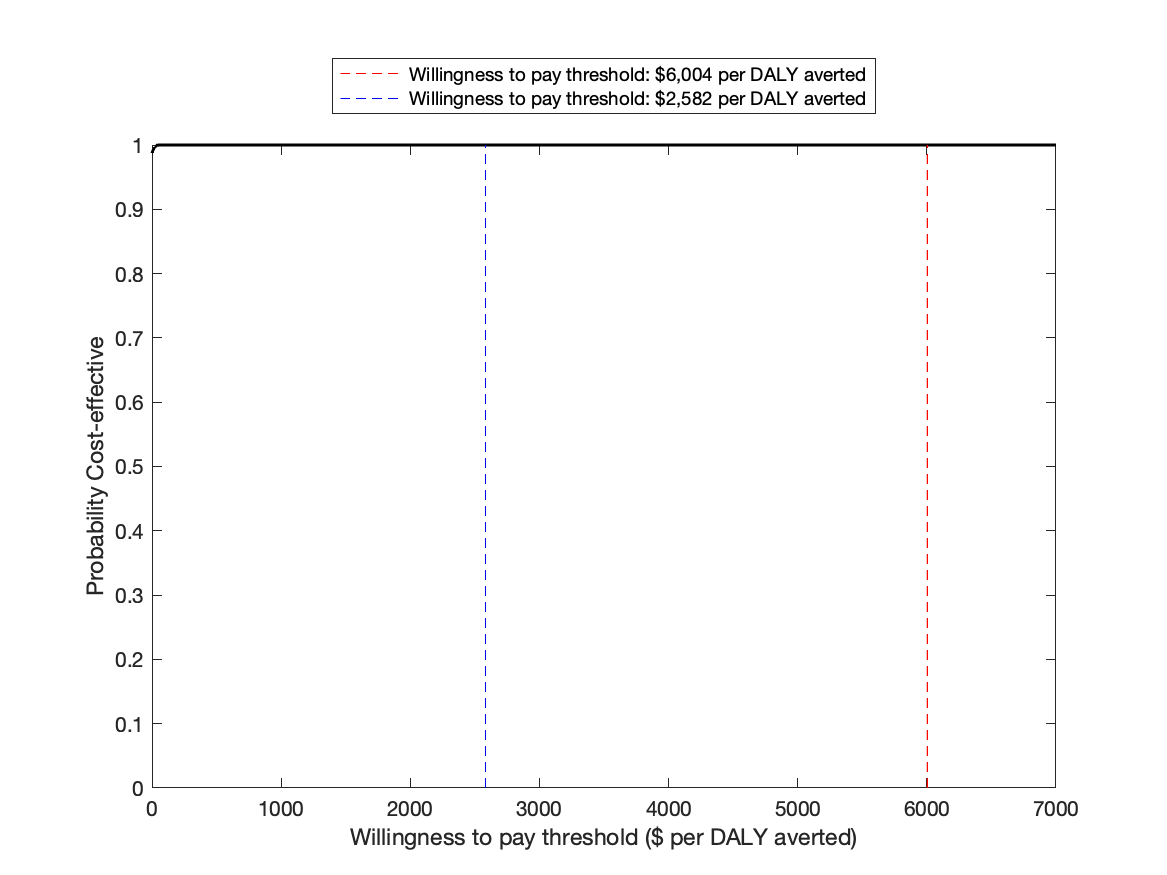


**Supplementary Figure 9:** *Cost-effectiveness acceptability curve for the Baseline cost-effectiveness analysis. Dashed lines show the WTP threshold of 1xGDP (red) and 0.43xGDP (blue).*

**Supplementary Table 2:** Results of uncertainty analyses. Parameters contributing to >=2% of variability shown.

| **% HIV infections averted** | | **DALYs averted** | | **Costs averted** | |
| --- | --- | --- | --- | --- | --- |
| **Parameter** | **% of variability (sign of correlation)** | **Parameter** | **% of variability (sign of correlation)** | **Parameter** | **% of variability (sign of correlation)** |
| Proportion of PrEP users that start early ART if seroconvert | 34.9% (+) | Proportion of PrEP users that start early ART if seroconvert | 36.6% (+) | Proportion of PrEP users that start early ART if seroconvert | 35.6% (-) |
| Reduction in frequency of commercial sex during Covid-19 lockdowns | 8.9% (-) | FSW condom use during commercial vaginal sex | 8.0% (+) | Reduction in frequency of commercial sex during Covid-19 lockdowns | 9.4% (+) |
| FSW condom use during commercial vaginal sex | 7.8% (-) | Reduction in frequency of commercial sex during Covid-19 lockdowns | 6.8% (-) | FSW condom use during commercial vaginal sex | 9.2% (-) |
| Average number of commercial partners that FSWs have | 7.3% (+) | Rate at which low risk females start commercial sex | 4.7% (+) | Prep efficacy when highly adherent | 5.6% (+) |
| Proportion of FSWs that have at least one main partner | 4.0% (-) | Average number of commercial partners that FSWs have | 4.0% (-) | Average number of commercial partners that FSWs have | 4.8% (-) |
| Proportion of male model entrants that are MSM | 3.9% (-) | ART recruitment rate among clients | 3.9% (+) | Rate at which low risk females start commercial sex | 3.9% (-) |
| Average number of main partners that FSWs have amongst those with at least one main partner | 3.6% (+) | Duration of the latent phase of infection | 3.3% (+) | Proportion of FSWs that have at least one main partner | 3.6% (+) |
| X111 | 2.4% (-) | ART recruitment rate among FSWs | 3.1% (-) | Average number of main partners that FSWs have amongst those with at least one main partner | 3.0% (-) |
| Prep efficacy when highly adherent | 2.3% (-) | ART recruitment rate among low risk males | 2.9% (-) |  |  |
|  |  | ART recruitment rate among low-risk females | 2.8% (-) |  |  |
|  |  | Proportion of FSWs that have at least one main partner | 2.8% (-) |  |  |
|  |  | Average number of main partners that FSWs have amongst those with at least one main partner | 2.1% (+) |  |  |

*
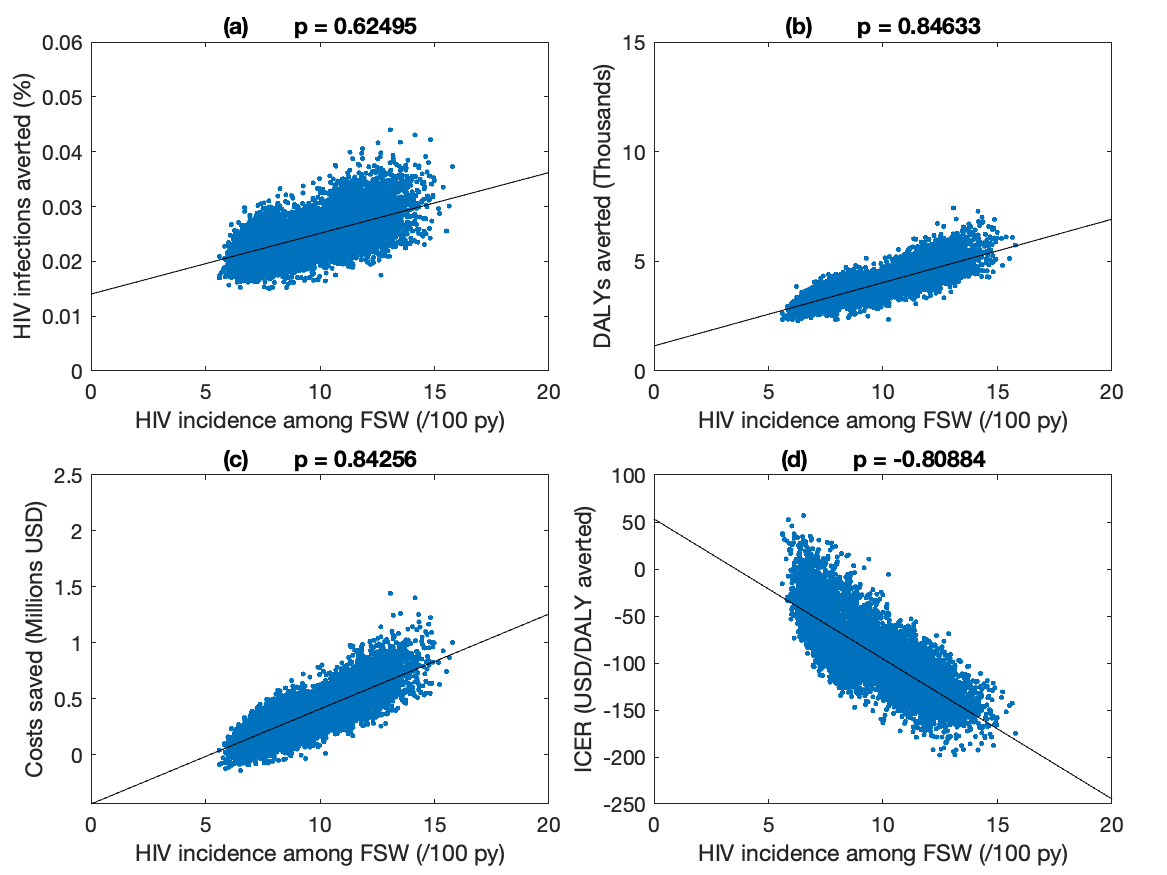
*

**Supplementary Figure 10:** Scatter plots showing the association between HIV incidence among FSW and the impact and cost-effectiveness of the PrEP intervention (2016-2020). (a) HIV infections averted over 2016-2040; (b) DALYs averted over 2016-2040; (c) costs saved over 2016-2040; (d) the ICER for PrEP. Black lines are the least-squares line. Correlation coefficients are presented in bold above each sub-figure.

# References

Chou, R., C. Evans, A. Hoverman, C. Sun, T. Dana, C. Bougatsos, S. Grusing and P. T. Korthuis (2019). "Preexposure Prophylaxis for the Prevention of HIV Infection: Evidence Report and Systematic Review for the US Preventive Services Task Force." JAMA **321**(22): 2214-2230.

Eakle, R., G. B. Gomez, N. Naicker, R. Bothma, J. Mbogua, M. A. Cabrera Escobar, E. Saayman, M. Moorhouse, W. D. F. Venter, H. Rees and T. D. P. Team (2017). "HIV pre-exposure prophylaxis and early antiretroviral treatment among female sex workers in South Africa: Results from a prospective observational demonstration project." PLoS Med **14**(11): e1002444.

Jaffer, M., N. Christofides, K. Hlongwane, K. Otwombe, M. Milovanovic, K. L. Hopkins, M. Matuludi, V. Mbowane, F. Abdullah, G. Gray, R. Jewkes and J. Coetzee (2022). "The HIV Cascade of Care and Service Utilisation at Sex Work Programmes Among Female Sex Workers in South Africa." AIDS Behav.

Janes, H., L. Corey, G. Ramjee, L. N. Carpp, C. Lombard, M. S. Cohen, P. B. Gilbert and G. E. Gray (2018). "Weighing the evidence of efficacy of oral PrEP for HIV prevention in women in Southern Africa." AIDS research and human retroviruses **34**(8): 645-656.

Johnson, L. F., P. Mulongeni, A. Marr and T. Lane (2018). "Age bias in survey sampling and implications for estimating HIV prevalence in men who have sex with men: insights from mathematical modelling." Epidemiol Infect **146**(8): 1036-1042.

Marrazzo, J. M., G. Ramjee, B. A. Richardson, K. Gomez, N. Mgodi, G. Nair, T. Palanee, C. Nakabiito, A. van der Straten, L. Noguchi, C. W. Hendrix, J. Y. Dai, S. Ganesh, B. Mkhize, M. Taljaard, U. M. Parikh, J. Piper, B. Masse, C. Grossman, J. Rooney, J. L. Schwartz, H. Watts, M. A. Marzinke, S. L. Hillier, I. M. McGowan, Z. M. Chirenje and V. S. Team (2015). "Tenofovir-based preexposure prophylaxis for HIV infection among African women." N Engl J Med **372**(6): 509-518.

Mukandavire, C., J. Walker, S. Schwartz, M. C. Boily, L. Danon, C. Lyons, D. Diouf, B. Liestman, N. L. Diouf, F. Drame, K. Coly, R. S. M. Muhire, S. Thiam, P. A. N. Diallo, C. T. Kane, C. Ndour, E. Volz, S. Mishra, S. Baral and P. Vickerman (2018). "Estimating the contribution of key populations towards the spread of HIV in Dakar, Senegal." J Int AIDS Soc **21 Suppl 5**: e25126.

Stone, J., C. Mukandavire, M. C. Boily, H. Fraser, S. Mishra, S. Schwartz, A. Rao, K. J. Looker, M. Quaife, F. Terris-Prestholt, A. Marr, T. Lane, J. Coetzee, G. Gray, K. Otwombe, M. Milovanovic, H. Hausler, K. Young, M. McIngana, M. Ncedani, A. Puren, G. Hunt, Z. Kose, N. Phaswana-Mafuya, S. Baral and P. Vickerman (2021). "Estimating the contribution of key populations towards HIV transmission in South Africa." J Int AIDS Soc **24**(1): e25650.

Toni, T., D. Welch, N. Strelkowa, A. Ipsen and M. P. Stumpf (2009). "Approximate Bayesian computation scheme for parameter inference and model selection in dynamical systems." J R Soc Interface **6**(31): 187-202.

Van Damme, L., A. Corneli, K. Ahmed, K. Agot, J. Lombaard, S. Kapiga, M. Malahleha, F. Owino, R. Manongi, J. Onyango, L. Temu, M. C. Monedi, P. Mak'Oketch, M. Makanda, I. Reblin, S. E. Makatu, L. Saylor, H. Kiernan, S. Kirkendale, C. Wong, R. Grant, A. Kashuba, K. Nanda, J. Mandala, K. Fransen, J. Deese, T. Crucitti, T. D. Mastro, D. Taylor and F. E.-P. S. Group (2012). "Preexposure prophylaxis for HIV infection among African women." N Engl J Med **367**(5): 411-422.
